# Transcriptomic analysis of *Litchi chinensis* pericarp during maturation with a focus on chlorophyll degradation and flavonoid biosynthesis

**DOI:** 10.1186/s12864-015-1433-4

**Published:** 2015-03-21

**Authors:** Biao Lai, Bing Hu, Yong-Hua Qin, Jie-Tang Zhao, Hui-Cong Wang, Gui-Bing Hu

**Affiliations:** State Key Laboratory for Conservation and Utilization of Subtropical Agro-Bioresources, College of Horticulture, South China Agricultural University, Guangzhou, 510642 Guangdong People’s Republic of China; Physiological Laboratory for South China Fruits, College of Horticulture, South China Agricultural University, Guangzhou, 510642 People’s Republic of China

**Keywords:** Litchi, Transcriptome, RNA-Seq, DGE (digital gene expression), Flavonoid biosynthesis, Chlorophyll degradation

## Abstract

**Background:**

The fruit of litchi (*Litchi chinensis*) comprises a white translucent edible aril surrounded by a pericarp. The pericarp of litchi has been the focus of studies associated with fruit size, coloration, cracking and shelf life. However, research at the molecular level has been limited by the lack of genomic and transcriptomic information. In this study, an analysis of the transcriptome of litchi pericarp was performed to obtain information regarding the molecular mechanisms underlying the physiological changes in the pericarp, including those leading to fruit surface coloration.

**Results:**

Coincident with the rapid break down of chlorophyll, but substantial increase of anthocyanins in litchi pericarp as fruit developed, two major physiological changes, degreening and pigmentation were visually apparent. In this study, a cDNA library of litchi pericarp with three different coloration stages was constructed. A total of 4.7 Gb of raw RNA-Seq data was generated and this was then *de novo* assembled into 51,089 unigenes with a mean length of 737 bp. Approximately 70% of the unigenes (34,705) could be annotated based on public protein databases and, of these, 3,649 genes were significantly differentially expressed between any two coloration stages, while 156 genes were differentially expressed among all three stages. Genes encoding enzymes involved in chlorophyll degradation and flavonoid biosynthesis were identified in the transcriptome dataset. The transcript expression patterns of the Stay Green (SGR) protein suggested a key role in chlorophyll degradation in the litchi pericarp, and this conclusion was supported by the result of an assay over-expressing *LcSGR* protein in tobacco leaves. We also found that the expression levels of most genes especially late anthocyanin biosynthesis genes were co-ordinated up-regulated coincident with the accumulation of anthocyanins, and that candidate MYB transcription factors that likely regulate flavonoid biosynthesis were identified.

**Conclusions:**

This study provides a large collection of transcripts and expression profiles associated with litchi fruit maturation processes, including coloration. Since most of the unigenes were annotated, they provide a platform for litchi functional genomic research within this species.

**Electronic supplementary material:**

The online version of this article (doi:10.1186/s12864-015-1433-4) contains supplementary material, which is available to authorized users.

## Background

Litchi (*Litchi chinensis* Sonn.), a member of the Sapindaceae, is an important fruit crop that is widely cultivated in tropical and subtropical areas of the world. The fruit are fleshy drupes with an edible aril surrounded by the pericarp, which has been the focus of many studies associated with fruit size, coloration, cracking and storability.

Substantial changes in color are often evident during fruit maturation and can provide an important indication of maturity and quality in many fruit species [1]. For example, in litchi a red color on the fruit surface is commercially desirable, although some litchi cultivars do not have a strong red coloration, mainly due to slow chlorophyll degradation in the pericarp. High concentrations of chlorophylls in the pericarp not only mask the red fruit surface color that is provided by anthocyanins, but also slow their biosynthesis [2]. This can be ameliorated to some extent during cultivation by the practice of bagging, which is known to enhance chlorophyll degradation in the litchi pericarp and to promote fruit pigmentation. Chlorophyll breakdown contributes to a stronger coloration during fruit ripening as the photosynthetic pigment absorbs much of the incident red light, thus reducing the degree of phytochrome control of anthocyanin biosynthesis [3]. Consequently, much higher irradiation is required for anthocyanin synthesis in green tissue compared with etiolated tissue. Recently, many steps in the chlorophyll degradation pathway in leaves undergoing senescence have been characterized using mutants with disrupted chlorophyll degradation [4] and chlorophyll catabolism enzymes (CCEs) and the stay green (SGR) protein have been shown to play a prominent role [5]. In addition, studies of kiwifruit (*Actinidia chinensis*) have indicated that genes involved in chlorophyll degradation, such as SGR2, are expressed at higher levels in golden fresh cultivar than in a green cultivar, resulting in earlier and more sustained chlorophyll degradation [6]. However, chlorophyll degradation in many fruit species is still poorly understood.

Anthocyanins are examples of flavonoids, the biosynthetic pathways of which have been extensively studied not only because they result in the production of red, blue and black plant pigments, but also in the contexts of their diverse roles in UV protection and pathogen defense, as well as their nutritional value in the human diet [7]. The red pericarp color of litchi fruit is known to result from anthocyanin accumulation [8], and the pericarp also contains an abundance of phenolic compounds (51 to 102 g kg^−1^ dry weight), which inhibit fat acid oxidation and act as free radical scavengers [9]. Of these, oligonol, a polyphenolic compound containing catechin-type monomers and short oligomers of proanthocyanidin, has been particularly noted to have health benefits [10]. However, pericarp browning, a phenomenon that can have a highly negative effect on fruit quality and shelf life, can be attributed to the oxidation of phenolics [11]. Thus, the study of the biosynthesis and metabolism phenolic compounds and flavonoids in the pericarp of litchi fruit has considerable potential commercial value for a number of reasons.

Studies to date have identified two basic classes of genes involved in flavonoid biosynthesis: structural genes of the flavonoid pathway that are common to a range of species, and regulatory genes that modulate the activity of the biosynthetic genes, thereby controlling the spatial and temporal accumulation of the pigments [7]. Examples of both classes of genes have been identified and characterized from many fruit species [8,12-15]. Anthocyanin biosynthesis in the pericarp of litchi shows cultivar, developmental and environmental associated variation [8,13] and both a structural gene (*LcUFGT*) and a transcription factor (*LcMYB1*) have been shown to play major roles in these differences [8,13]. However, only one gene member in each multigene family of the structural genes in litchi flavonoid biosynthesis has been reported to date and transcription factors (TFs) that have been shown to interact with members of the MYB TF family to regulate the biosynthesis of flavonoids, such as basic helix-loop-helix (bHLH) TFs and the WD40 protein [16] have not yet been characterized in litchi.

Chlorophyll degradation, anthocyanin accumulation, increase in membrane permeability, and cell wall disassembly have all been suggested to coincide with the onset of litchi maturation [17], but the specific factor(s) that triggers this important transition in litchi fruits is still unknown. Previous studies have demonstrated a differential effect of ABA and ethylene on litchi pericarp coloration. ABA is suggested to be more important in anthocyanin synthesis, while ethylene thought to have a more significant role in chlorophyll degradation [18]. However, the molecular mechanisms that control the maturation of these non-climacteric fruits remains to be elucidated.

A particularly limiting factor in this regard, and indeed in advancing litchi fruit research at the molecular level has been the scarcity of gene sequence information, and even the most comprehensive studies to date typically have considered only a few hundred gene sequences [19]. Recent advances in RNA-Seq transcriptome profiling technologies based on NGS (next-generation sequencing) have enabled a powerful platform to address questions involving complex patterns of gene expression such as those listed above. Moreover, RNA-Seq analysis facilitates research using species without a published genome sequence, such as litchi, by providing massive sequence data sets for molecular marker development, gene discovery, transcriptional analysis, and pathway enrichment analysis. In this context digital gene expression (DGE) is a tag-based transcriptomic sequencing approach in which the expression levels of genes can be measured by counting the number of expressed sequence tags (ESTs) derived from each specific gene, enabling the assessment of expression levels between samples or during developmental processes [20,21].

In this study, a cDNA library of litchi pericarp was constructed and 51,089 unigenes were assembled based on Illumina RNA-seq data. A total of 4.7 Gb was used for *de novo* transcriptome assembly, resulting in a comprehensive data set for the identification of genes corresponding to the major metabolic pathways in the pericarp of litchi. Global gene expression profiles, focusing mainly on chlorophyll degradation and flavonoid biosynthesis, during fruit development were analyzed using a DGE strategy. In addition, structural and regulatory genes associated with pericarp coloration were identified. This data set will serve as a platform to advance the understanding of the regulatory mechanisms underlying the development of litchi fruit and possibly those of other non-climacteric fruits.

## Results

### Chlorophyll and flavonoid levels in the pericarp of three developmental stages of litchi fruit

**Figure 1:**
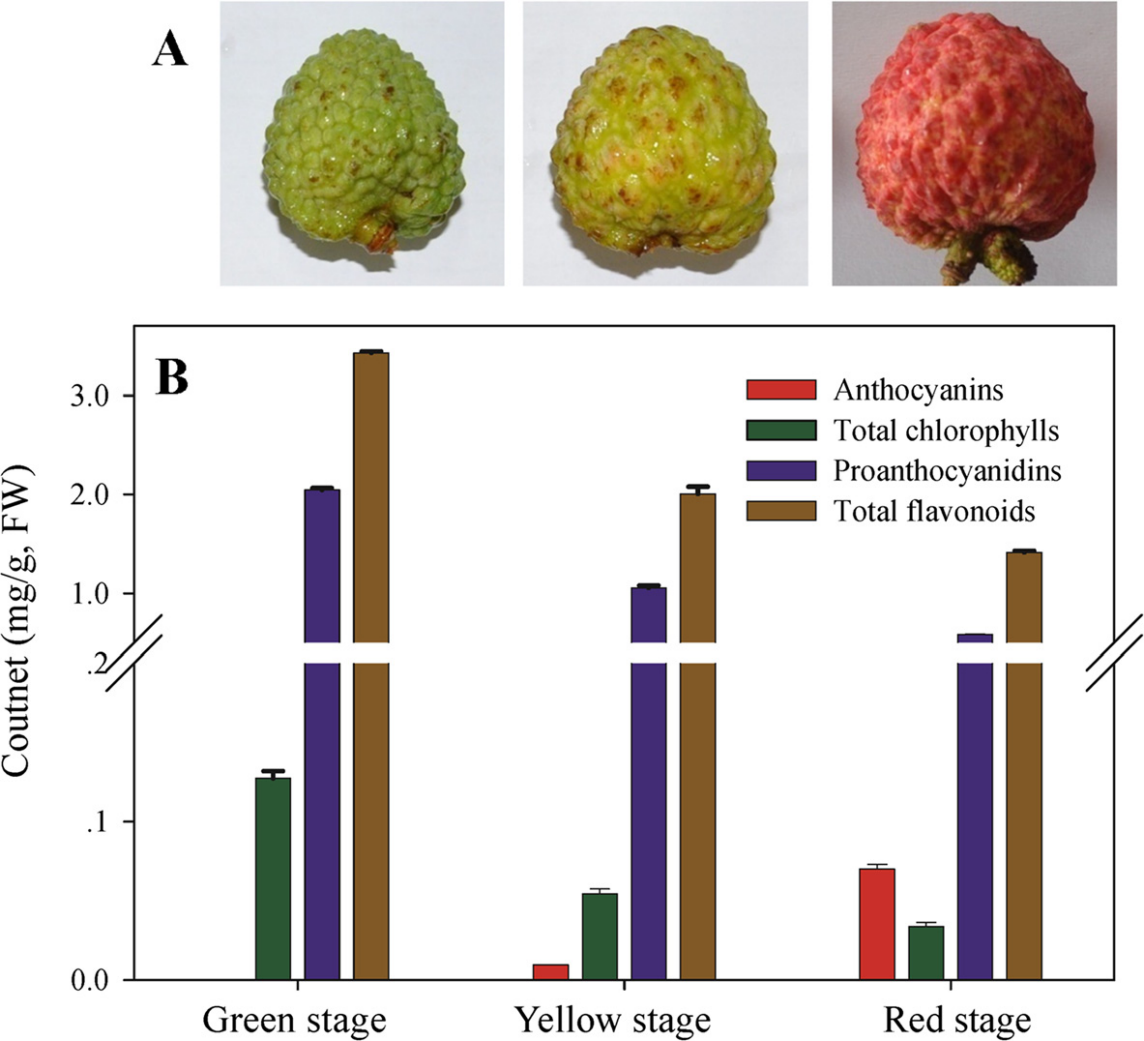
**Images of litchi fruits and pigment contents in the pericarp. (A)** Images of litchi fruits at different coloration stages. **(B)** Contents of total chlorophylls, total flavonoids, proanthocyanidins and anthocyanins in the pericarp of three coloration stages. The vertical bars represent the standard error of triplicate experiments.

As the litchi fruit developed, two major physiological changes, degreening and pigmentation were visually apparent (Figure 1A) and these coincided with changes in the abundance of chlorophyll, total flavonoids, proanthocyanidins, and anthocyanins in the pericarp (Figure 1B). We determined that more than half of the chlorophyll breaks down within 10 days in parallel with an obvious degreening at 62 days after anthesis (DAA). Chlorophyll levels then continued to decrease but at a reduced rate as fruit developed toward full maturity (72 DAA). Total flavonoid and proanthocyanidin levels decreased during fruit development. No anthocyanins were detected in the green pericarp (52 DAA); however, low levels accumulated in the yellow pericarp and the content increased substantially through the rest of development, giving rise to a characteristic red pigmentation.

### Library construction

After removal of adaptor sequences, ambiguous reads and low-quality reads (Q20 < 20), a total of 51,746,546 high-quality filtered reads of 75-bp length, comprising 4,657,189,140 nucleotides (4.7 Gb) was obtained. All high-quality reads were *de novo* assembled into 57,361 contigs (756 bp average), with a N50 of 1,156 bp. After paired-end joining and gap-filling, these contigs were then assembled into 51,089 unique sequences with a mean size of 737 bp. The size distributions of these contigs and unigenes are shown in Additional file 1.

### Annotation of predicted proteins

**Figure 2:**
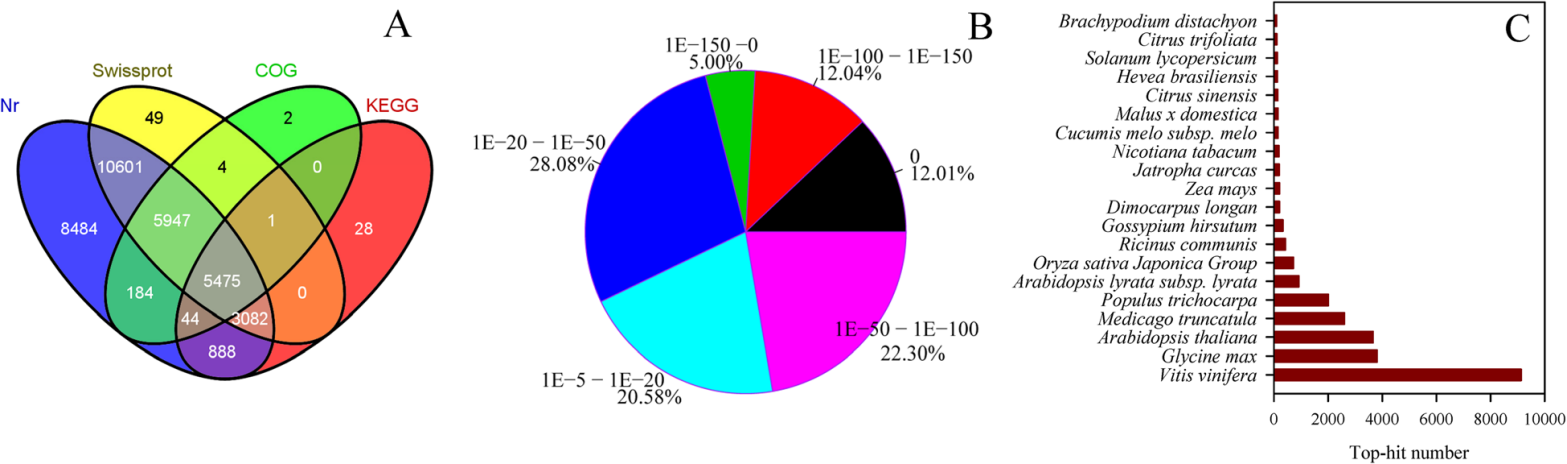
**Outcome of homology search of litchi unigenes against the nr database. (A)** Venn diagram of unigene numbers annotated by BLASTx with an E-value threshold of 10^−5^ against protein databases. The numbers in the circles indicate unigenes numbers annotated by single or multiple databases. **(B)** E-value distribution of the top BLAST hits for each unique sequence. **(C)** Species distribution of the top BLAST hits for all homologous sequences.

Approximately 34,789 unique sequences were annotated based on BLASTx (cut-off E-value 10^−5^) searches of four public databases: the NCBI non-redundant (nr) database, the Swiss-Prot protein database, the Kyoto Encyclopedia of Genes and Genomes (KEGG) database, and the COG database (Figure 2A). Of these, 34,705 unique sequences were annotated with reference to the nr database, while 5,475 unigenes were annotated using the other databases.

Based on the nr annotations, 29% of the annotated sequences had ‘very strong homology’ (E-value < 10^−100^), 22% had ‘strong homology’ (10^−100^ < E-value < 10^−50^) and 49% had ‘homology’ (10^−50^ < E-value < 10^−5^), to available plant sequences (Figure 2B). 32% of the unique sequences had top matches to sequences from grape (*Vitis vinifera*), with additional hits to soybean (*Glycine max*) sequences (14%), *Medicago truncatula* sequences (9%), *Populus trichocarpa* sequences (7%), and *Arabidopsis thaliana* sequences (3%) (Figure 2C).

A total of 34,436 CDS (coding sequences) were identified using BLASTx against the databases mentioned above, and an additional 1,423 unigenes were predicted using the ESTScan program, so the direction and region of these genes could be determined and their sequences be translated into peptide sequences, of which 8,715 (24%) had a length over 300 AA. The detailed length distribution is shown in Additional file 1.

### Functional classification

**Figure 3:**
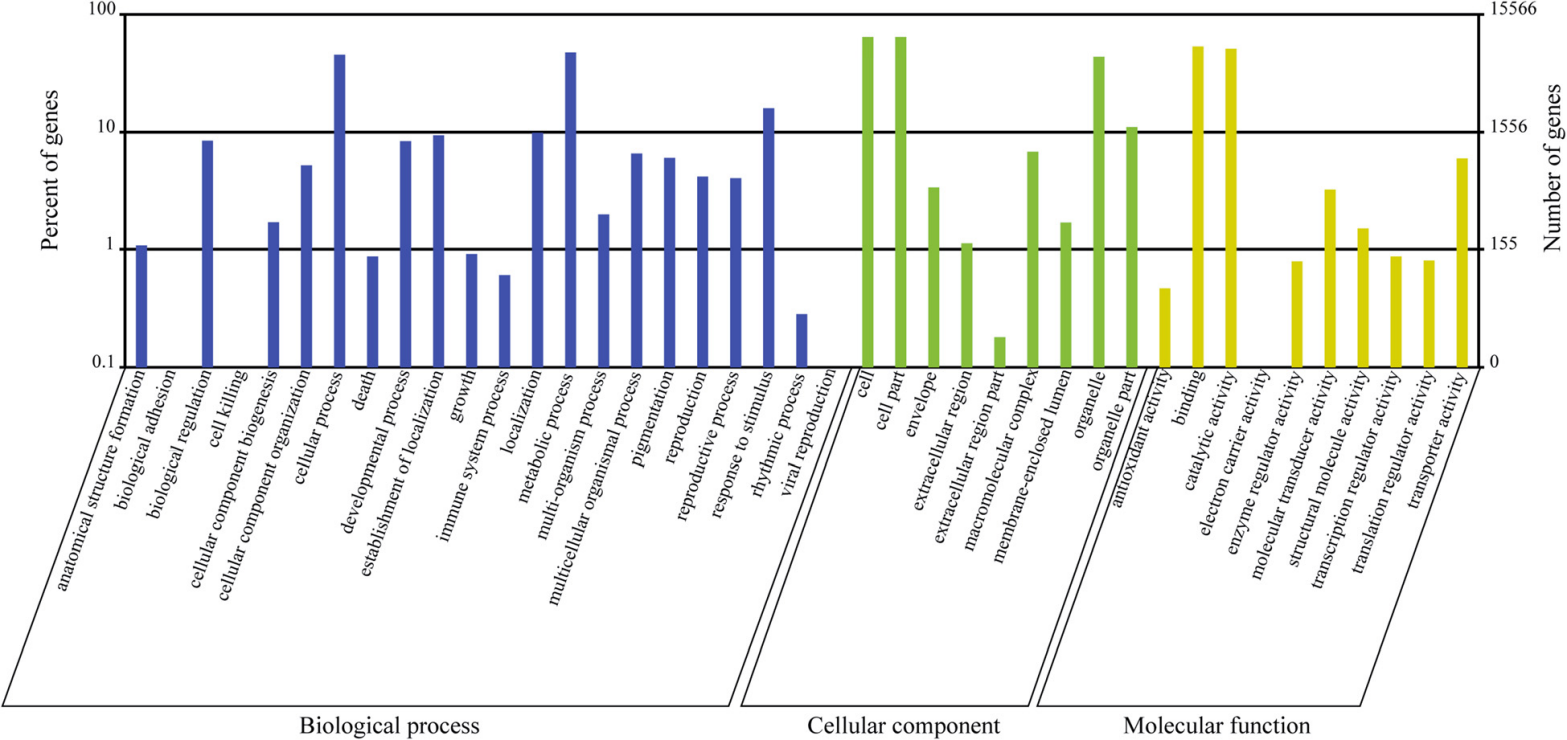
**Histogram of gene ontology (GO) classifications for litchi pericarp transcripts.** The unigenes corresponded to three main categories: ‘biological process’, ‘cellular component’ and ‘molecular function’. The left and right-hand y-axes indicate the percentage and number of annotated unigenes, respectively.

The GO, KEGG and COG databases were used to classify the functions of the predicted litchi pericarp unigenes. Approximately 15,566 unigenes were classified into three main categories: ‘biological process’, ‘cellular component’ and ‘molecular function’ (Figure 3). Within the ‘cellular component’ category, a large number of unigenes were annotated as ‘cell and organelle’, while the major groups within the ‘biological process’ category were ‘metabolic process’ (7,375 unigenes, 47%), ‘cellular process’ (7,069 unigenes, 45%) and ‘response to stimulus’ (2,479 unigenes, 16%). In the ‘molecular function’ category, ‘binding’ (8,303 unigenes, 53%) and ‘catalytic activities’ (7,933 unigenes, 51%) were the two most abundant subcategories.

A total of 9,518 unigenes were mapped onto 123 KEGG pathways. The maps with highest unigene presentation were ribosome (ko03010, 322 unigenes), followed by RNA transport (ko03013, 309 unigenes), plant hormone signal transduction (ko04075, 264 unigenes) and protein processing in the endoplasmic reticulum (ko04141, 262 unigenes). The pathways with highest presentation were purine metabolism (Ko00230, 252 unigenes), starch and sucrose metabolism (Ko00500, 224 unigenes), pyrimidine metabolism (Ko00240, 173 unigenes) and phenylpropanoid biosynthesis (ko00940, 147 unigenes) (Additional file 2). Lastly, the COG database is used to classify orthologous proteins. Every protein in the COG database is assumed to have evolved from an ancestor and the database includes the amino acid sequences for protein encoding genes derived from complete genome sequences as well as the evolutionary relationships of bacteria, algae and eukaryotes [22]. These data are therefore useful for protein classification and studies of evolutionary rates. As shown in Additional file 3, group K (Transcription factors), group L (Replication recombination and repair), group O (Post-translational modification, protein turnover, and chaperones) and group T (Signal transduction mechanisms) are the four most abundant groups represented in the litchi data set, indicating that pericarp maturation involves substantial amounts of both transcriptional and post-translational regulation of gene expression and function.

### DGE library sequencing and sequence mapping to the transcriptome database

Changes in gene expression in the pericarp of the three fruit developmental stages were analyzed using RNA-Seq. Three DGE libraries (green stage, yellow stage, and red stage) were sequenced to generate approximately 6 million filtered reads per library. The total number of mapped reads in each library ranged from 4.2-5.0 million and the percentage of these reads mapped to the de novo assembled transcriptome ranged from 73-83%. Of these the number of uniquely matched reads ranged from 3.6-4.1 million (Table 1). The random distribution of reads matching the reference genes was assessed and most reads were found to be evenly distributed throughout the transcriptome (Additional file 4). All three RNA-Seq libraries showed similar patterns of distribution of unique reads among the different read abundance categories (Additional file 5).

**Table 1:** **Summary of read numbers based on the RNA-Seq data from the pericarp of litchi (cv. Nuomici) during the green, yellow and red stages of maturation**

**Summary**	**Green**	**Yellow**	**Red**
Total reads	6,102,035	5,859,401	6,118,442
Q20 Percentage	96.8%	96.2%	96.9%
Total mapped reads	4,729,816	4,298,029	5,049,453
Total mapped reads/total clean reads	78%	73%	83%
Perfect match	3,005,300	2,693,246	3,358,974
Perfect match reads/total clean reads	49%	46%	55%
≤2 bp Mismatch	1,724,516	1,604,783	1,690,479
≤2 bp Mismatch reads/total clean reads	28%	27%	28%
Unique match	3,617,316	3,170,908	4,128,578
Unique match reads/total clean reads	59%	54%	68%
Multi-position match	1,112,500	1,127,121	920,875
Multi-position match reads/total clean reads	18%	19%	15%
Total unmapped reads	1,372,219	1,561,372	1,068,989
Total unmapped reads/total clean reads	23%	27%	18%

### Differential gene expression between three stages of coloration

**Figure 4:**
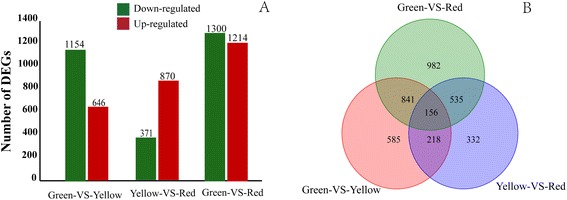
**Differential gene expression profiles based on the library of the three coloration stages. (A)** The numbers of up- and down-regulated genes in comparisons of the red-VS-yellow, red-VS-green, and yellow-VS-green fruit samples. **(B)** Venn diagram showing the comparison of differentially expressed genes between any two stages of the litchi pericarp.

Differences in gene expression in the pericarp at three coloration stages (green-VS-yellow, yellow-VS-red, and green-VS-red) were assessed and DEGs were identified by pairwise comparisons of the three libraries with the expression fold (log_2_Ratio ≥ 1) and false discovery rate (FDR ≤ 10^−3^) as the thresholds (Figure 4A). A total of 3,649 genes were found to be significantly differentially expressed in the pair-wise comparisons between any two stages, with 1,800 DEGs (1,154 down-regulated and 646 up-regulated) between the green and yellow libraries (Additional file 6). A total of 1,241 DEGs were detected between the yellow and red libraries, with 371 down-regulated and 870 up-regulated (Additional file 7). Finally, the greatest number of differentially expressed genes occurred between the green and red libraries, with 1,300 down-regulated and 1,214 up-regulated (Additional file 8). Of all the DEGs, 156 genes were found to be significantly differentially expressed among all three coloration stages (Figure 4B).

**Figure 5:**
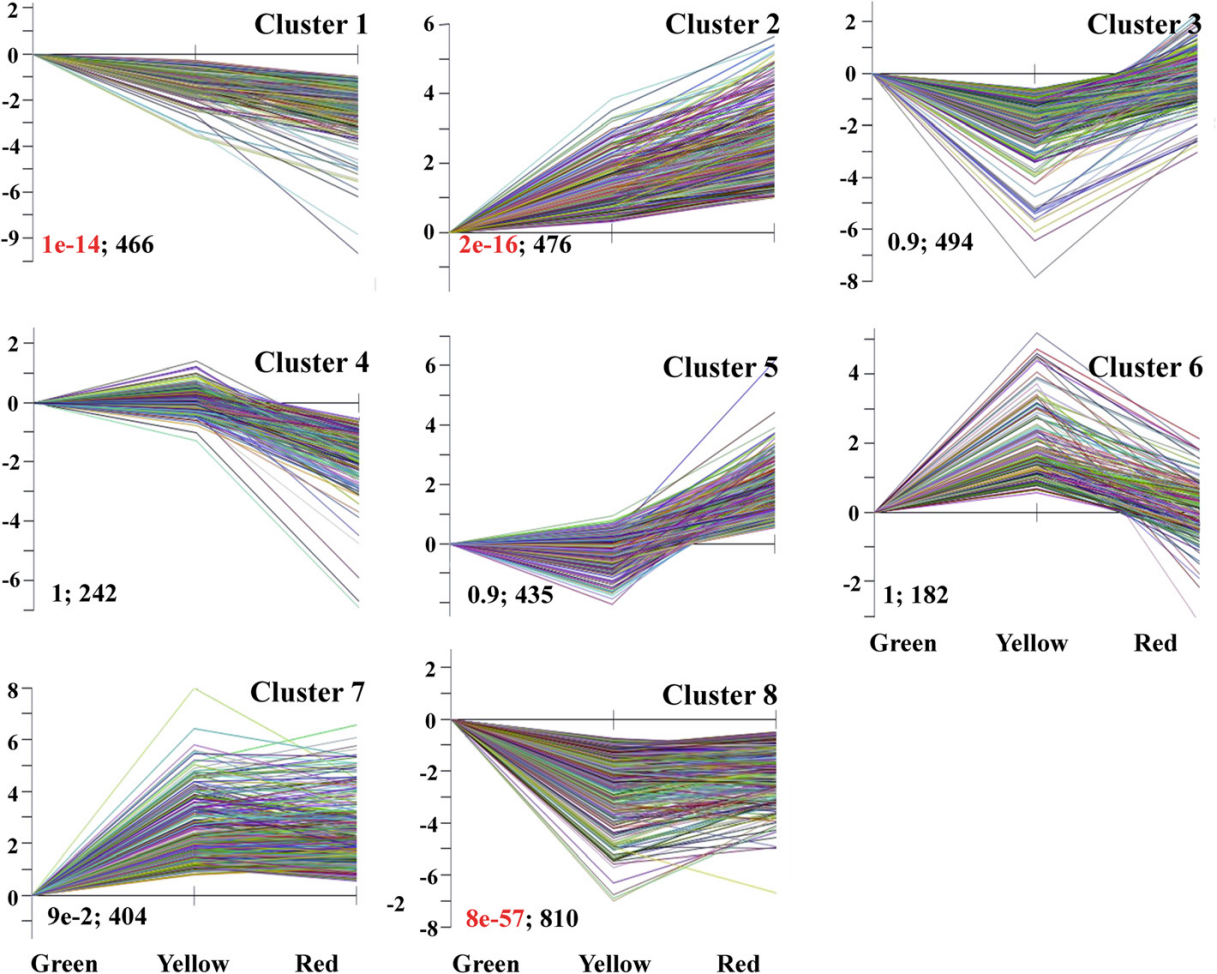
**Clustering results of time-course data from RNA-Seq by STEM analysis.** In the image each box corresponds to one of the model temporal expression profiles. The clusters with an asterisk had a statistically significant number of genes assigned. The number in the top right-hand corner of a profile box is the cluster number. The number before a semicolon at the bottom of a cluster box is the P-value for the genes assigned to a profile compared with what was expected. The number after the semicolon is the number of genes assigned to the profile.

We used the STEM (Short Time-series Expression Miner) software package to cluster our filtered DGE data into 8 distinct expression patterns (Figure 5) to identify genes with similar expression patterns, and that might therefore be functionally correlated. Clusters 1 to 8 include a broad range of genes belonging to the ‘metabolic processes’, ‘cellular processes’, ‘responses to stimulus’ and ‘biological regulation’ categories. The ‘biological process’ distribution frequency was calculated for each cluster to identify differences in the distribution of genes among three coloration stages (Additional file 9). Among the 8 clusters, clusters 1, 2 and 8 had a statistically significant number of genes assigned. Cluster 1 contained genes negatively modulated during pericarp coloration, while cluster 2 contained genes with a positive correlation. Cluster 8 contained 810 genes, whose expression levels decreased sharply from the green to the yellow stage, but subsequently increased significantly from the yellow to the red stage.

**Figure 6:**
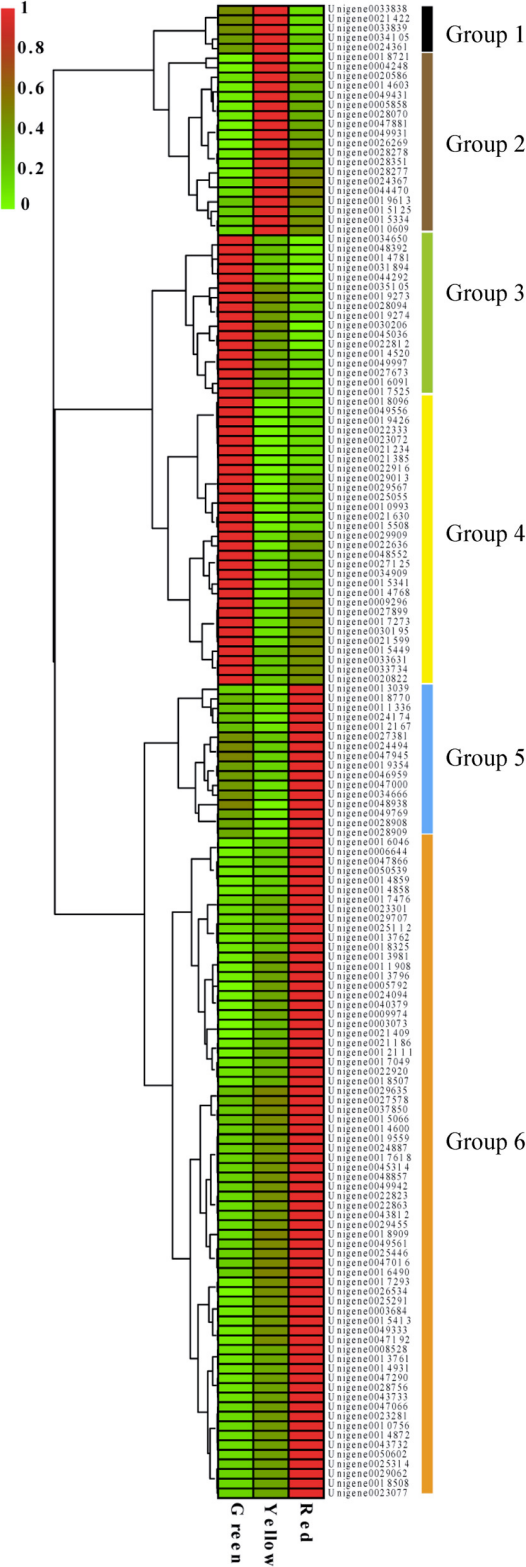
**Hierarchical clustering analysis of differentially-expressed genes (DEGs) during litchi pericarp maturation.**

The 156 DEGs shared between all three coloration stages were further analyzed (Additional file 10) and arranged into 6 groups (Figure 6), which collectively showed three different patterns. Genes in groups 1 and 2 were up-regulated from the green to the yellow stage, but down-regulated from the yellow to the red stage. Group 1 contained 5 genes, including a beta-galactosidase, a peroxidase and a thioredoxin gene, while group 2 contained 19 genes (12%), which encoded proteins including cytochrome P450, cysteine-rich receptor-like protein kinase, WRKY domain class TFs and sesquiterpene synthase. In groups 3 and 4, genes were highly expressed at the green stage but significantly down-regulated at the yellow and the red stages. Group 3 comprised 17 genes (11%), which encoded proteins such as laccase, tonoplast intrinsic protein (TIP), TCP like transcription factor and vegetative storage protein and group 4 was the second largest group, with 30 (19%) genes, including cellulose synthase, cytochrome P450, laccase, polygalacturonase, beta-1,4-xylosyltransferase and lipoxygenase. The genes in groups 5 and 6 belonged to the third developmental pattern, with a low expression at the green and the yellow stages but a dramatic up-regulation at the red stage. Group 5 genes included sesquiterpene synthase, terpene synthase, homogentisate geranylgeranyl transferase and an AP2/ERF domain-containing TF. The largest group (group 6) comprised 69 (44%) genes, including those encoding proteins associated with anthocyanin biosynthesis and transport such as UDP-flavonoid glucosyl transferase (UFGT), chalcone synthase (CHS), glutathione *S*-transferase (GST), an anthocyanin related MYB TF, stress response proteins such as a TIFY family gene, CMPG1, metallothionein-like protein, and allene oxide cyclase, fruit ripening related proteins, such as an ethylene-responsive TF and gretchen hagen 3 (GH3), and post-transcriptional modification and signal transduction proteins such as MAPK, MAPKKK, E3 ubiquitin-protein ligase, a GRAS family TF and the REM1.3 remorin protein. Six NAC TFs were also present in this group, indicating an important role of this TF family in litchi pericarp maturation, as were a R2R3-MYB TF, a WD40-repeat protein, a bHLH protein, and a CBF TF. We also identified 25 transcripts that had no functional annotation in any database.

### Genes involved in chlorophyll degradation

**Figure 7:**
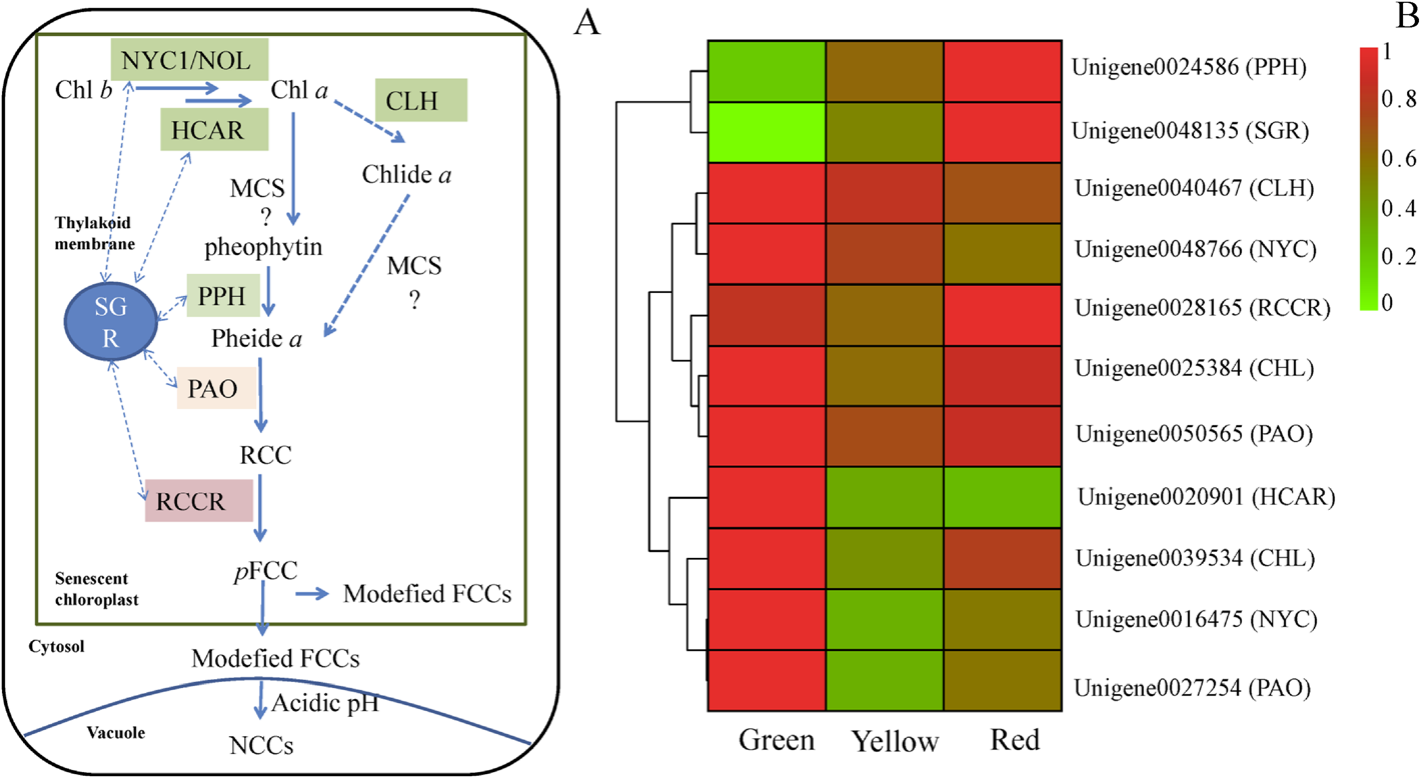
**The pathway of chlorophyll breakdown and expressions of chlorophyll breakdown genes. (A)** The PAO pathway of chlorophyll breakdown. **(B)** A heat map of the expressions of genes for chlorophyll breakdown in the pericarp during coloration. Chl, chlorophyll; CLH, chlorophyllase; HCAR, hydroxy-Chl a reductase; MCS, metal chelating substance; NCCs, nonfluorescent Chl catabolites; NOL, NYC1-like; NYC1, non-yellow coloring 1; PAO, pheide a oxygenase; pFCC, primary fluorescent Chl catabolite; Pheide, pheophorbide; PPH, pheophytinase; RCC, red Chl catabolite; RCCR, RCC reductase; SGR, stay-green protein.

One of the most distinct developmental changes in the pericarp of litchi is degreening, which results from rapid chlorophyll degradation. In land plants, chlorophyll is broken down to colorless linear tetrapyrroles in a highly conserved multi-step pathway termed the ‘PAO pathway’ (Figure 7A); so named because the opening of the chlorin macrocycle present in chlorophyll is catalyzed by pheophorbide a oxygenase (PAO) [4]. In the present study, eleven candidate genes related to chlorophyll degradation were identified from the litchi pericarp transcriptome database, including genes encoding chlorophyll catabolic enzymes, non-yellow coloring (NYC), chlorophyllase (CLH), hydroxy-Chl a reductase (HCAR), pheophytinase (PPH), pheide a oxygenase (PAO), and red Chl catabolite reductase (RCCR), and a stay green protein (SGR). The expression patterns were showed in Figure 7B. The expressions of *Unigene 0024586* (*PPH*) and *Unigene 0048135* (*SGR*) were similar, with the lowest expression at the green stage, moderate expression at the yellow stage, and highest expression at the red stage. The opposite pattern was seen for *Unigene 0020901* (*HCAR)*, whose expression decreased during pericarp coloration. For *NYC*, *CLH* and *PAO*, more than one gene was identified and the different gene family members displayed different expression patterns in all cases. The expression of *Unigene 0039534* (*CLH)*, *Unigene 0016475* (*NYC*), and *Unigene 0027254* (*PAO*) decreased sharply from the green to the yellow stage before showing up-regulation as fruit reached full maturity. The expression of the remaining genes belonging to these families remained relative stable throughout fruit coloration.

**Figure 8:**
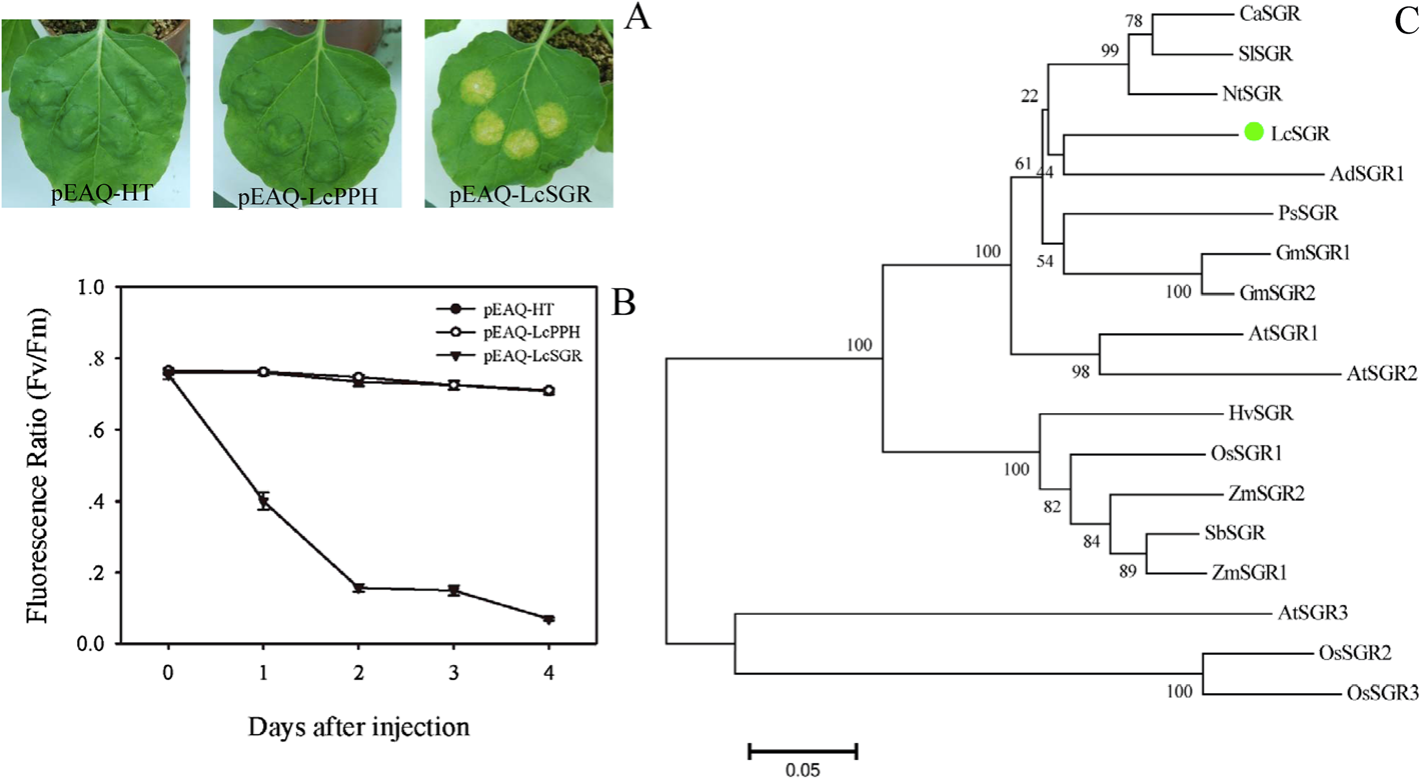
**Phylogenetic relationship of stay green (SGR) proteins and transient assays of LcSGR. A**. Transient assays of *LcSGR* in *Nicotiana benthamiana* leaf show the induction of substantial chlorophyll loss. **B**. A Loss of chlorophyll fluorescence due to chlorophyll degradation using a chlorophyll fluorometer. **C**. Phylogenetic relationship between LcSGR and other SGRs proteins from other plant species.

In this current study, the expression of *Unigene 0024586* (*PPH*) and *Unigene 0048135* (*SGR*) correlated with the degradation of chlorophylls during pericarp coloration. Transient expression of litchi *SGR* and *PPH* in *Nicotiana benthamiana* leaves showed different results. Distinct chlorophyll lost and a sharp decrease of chlorophyll fluorescence ratio from 0.78 to 0.15 were observed in *N. benthamiana* leaves transiently expressing litchi *SGR*, while no obvious degreening was noticed upon transient expression of litchi *PPH* (Figure 8A-B). Previous reports have indicated that *SGR* is essential for the initiation of chlorophyll breakdown in plastids by recruiting chlorophyll catabolism enzymes in senescing chloroplasts [4,5]. For example, after transient expression of kiwifruit (*Actinidia deliciosa*) *PPH1*, *PAO1*, *SGR1* or *SGR2* in expanding *N. benthamiana* leaves, only *SGR1* and *SGR2* caused chlorophyll degradation [6]. Phylogenetic analysis indicated that litchi SGR is closely related to AdSGR1*,* the kiwifruit SGR1 protein (Figure 8C). We conclude that, similar to AdSGR1, litchi SGR is a key senescence-induced chlorophyll degradation regulator.

### Genes related to flavonoid biosynthesis

**Figure 9:**
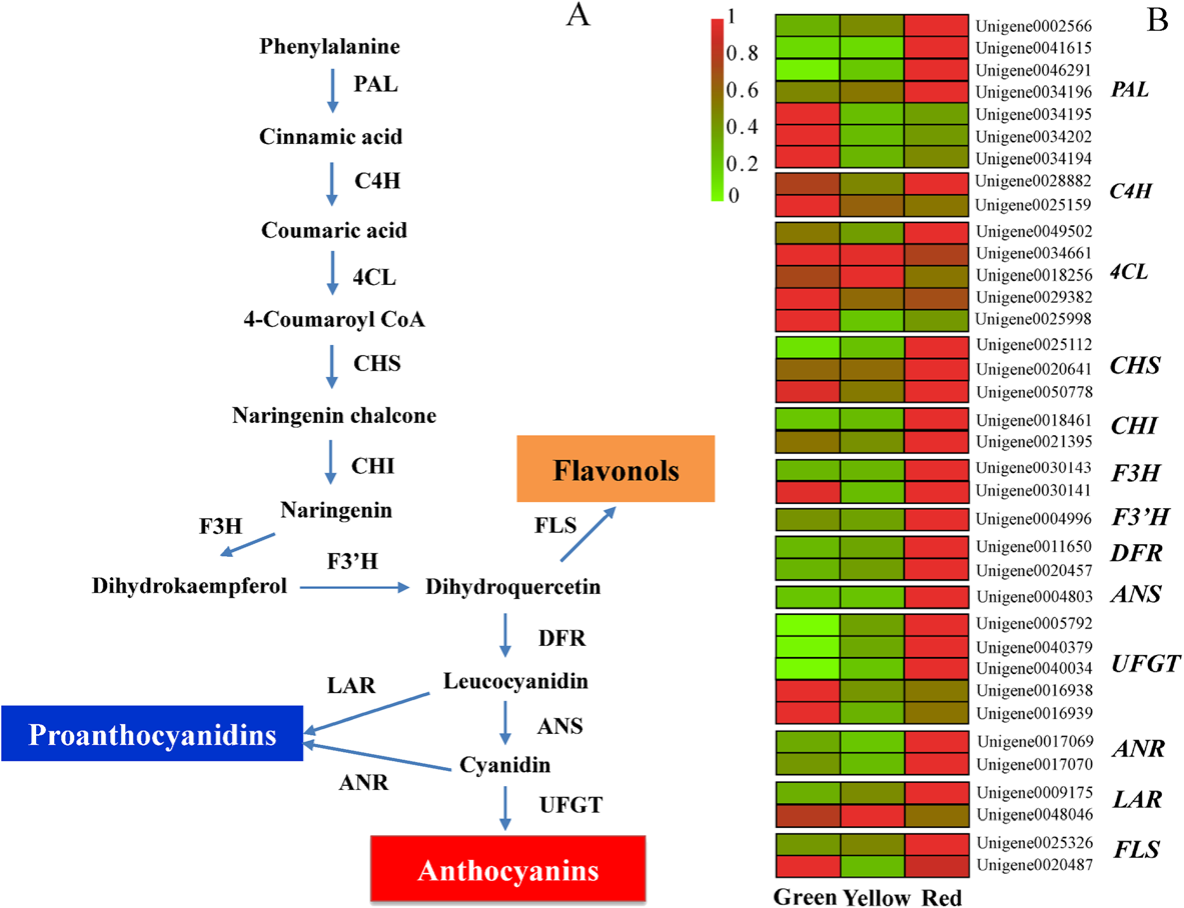
**Simplified scheme (A) and a heat map of the expression of genes (B) related to flavonoid biosynthesis.** Enzyme names are abbreviated as follows; PAL, phenylalanine ammonia lyase; C4H, cinnamic acid 4-hydroxylase; 4CL, 4 coumarate CoA ligase; CHS, chalcone synthase; CHI, chalcone isomerase; F3H, flavanone 3-hydroxylase; F3′H, flavanone 3′-hydroxylase; DFR, dihydroflavonol reductase; FLS, flavonol synthase; ANS/LDOX, anthocyanidin synthase/leucoanthocyanidin dioxygenase; UFGT, UDP-flavonoid glucosyltransferase; ANR, anthocyanidin reductase; and LAR, leucoanthocyanidin reductase. Enzyme names, unigene IDs and expression patterns are indicated on the right of each step.

Flavonoid biosynthesis is a highly studied plant secondary metabolism and thirteen enzymes are known to be involved in this pathway (Figure 9A). In this current study, we identified 35 candidate transcripts participating in each step of the flavonoid biosynthesis pathway (Figure 9B). Two distinct expression patterns were noticed among the seven *phenylalanine ammonia lyase* (*PAL*) genes. The transcription levels of *Unigene 0002566*, *Unigene 0041615*, *Unigene 0046291* and *Unigene 00034196* were low in green pericarp, but highly expressed in the red pericarp. The opposite expression pattern was observed for *Unigene 0034195*, *Unigene 0034194* and *Unigene 0034202* and two genes (*Unigene 0028882* and *Unigene 0025159*) corresponding for *cinnamate 4-hydroxylase* (*C4H*) displayed different expression patterns, with the expression level of the former being approximately 100 times higher than that of the latter during fruit coloration. Moreover, the highest expression of *Unigene 0028882* was detected in red pericarp while that of *Unigene 0025159* was detected in green pericarp. Five *4CL* genes were identified, of which the expression of *Unigene 0049502* was lowest at the yellow stage and highest at the red stage, while highest expression of *Unigene 0025998* occurred at the green stage and the remaining gene family members showed relatively constant expression during coloration. Three *chalcone synthase* (*CHS*) genes (*Unigene 0025112*, *Unigene 0020641* and *Unigene 0050778*) were identified in the litchi pericarp transcriptome. The nucleotide sequence of *Unigene 0020641* showed 99% identity with that of a previously reported litchi *CHS* gene, *LcCHS* (GU288820.1) [8]. Up-regulation of *Unigene 0025112* and *Unigene 0020641* expression was evident during coloration, while the expression level of *Unigene 0050778* was the lowest of the three CHS genes and a change during developmental was less apparent. Two genes (*Unigene 0018461* and *Unigene 0021395*) encoding chalcone isomerase (CHI) were also identified, both of which showed highest the expression at the red stage. *Flavanone 3-hydroxylase* (*F3H*, *Unigene 0030143*) and *flavanone 3′-hydroxylase* (*F3′H, Unigene 0004996*) had similar expression patterns and were down-regulated during the transition from green to yellow pericarp and then further up-regulated as the fruit reached maturity. Another *F3H* (*Unigene 0030141*) showed low expression at the yellow stage but high expression at the green and red stages.

The flavonoid biosynthesis pathway is usually divided into two parts: the early and the late sections [23]. The early section lead to the formation of the dihydro-flavonols, comprising PAL, C4H, 4CL, CHS, CHI and F3H, while the late stages of the flavonoid biosynthesis pathway include three key enzymes dihydroflavonol reductase (DFR), anthocyanidin synthase (ANS) and UDP-flavonoid glucosyltransferase (UFGT). Two of the DEGs, *Unigene 0011650* and *Unigene 0020457* encode predicted DFR and their expression levels increased during coloration. Only one gene (*Unigene 0004803*) encoding an ANS was found, and its expression was slightly up-regulated from the green to the yellow stage and then substantially up-regulated from the yellow to the red stage. Five genes encoding UFGT were identified. Of these, *Unigene 0005792*, *Unigene 0040379* and *Unigene 0040034* showed >97% identity to the known litchi UDP-glucose: flavonoid glucosyltransferase sequences in public database (HQ402914.1) [8]. However, the rest two unigenes (*Unigene 0016938* and *Unigene 0016939*) showed around 70% identity to pear UDP-galactose: flavonoid galactosyltransferase sequence (GU390548.1) [24]. Increasing expression that correlated with the accumulation of anthocyanins in three transcripts (*Unigene 0005792*, *Unigene 0040379* and *Unigene 0040034*) was noticed, while the expressions of other two *UFGT* (*Unigene 0016939* and *Unigene 0016938*) were highest at the green stage.

Genes encoding enzymes involved in other subgroups of flavonoid end products were also investigated. For example, four genes specifically participating in proanthocyanidin biosynthesis, encoding leucoanthocyanidin reductase (LAR, *Unigene 0009175* and *Unigene 0048046*) and anthocyanidin reductase (ANR, *Unigene 0017069* and *Unigene 0017070*), were identified in the litchi database. *Unigene 0009175* was up-regulated during fruit development, while the expression of *Unigene 0048046* was up-regulated slightly early in ripening, but decreased during the transition from the yellow to the red stage. The two *ANR* genes showed same expression pattern, with decreasing transcript accumulation from the green to the yellow stage but a substantial up-regulation from yellow to red pericarp. Two flavonol synthase genes, *Unigene 0020487* and *Unigene 0025326*, were also identified: the expression of the latter increased during fruit maturation while the expression of the former was lowest at the yellow stage.

### Coefficient analysis between RNA-Seq and real-time PCR

**Figure 10:**
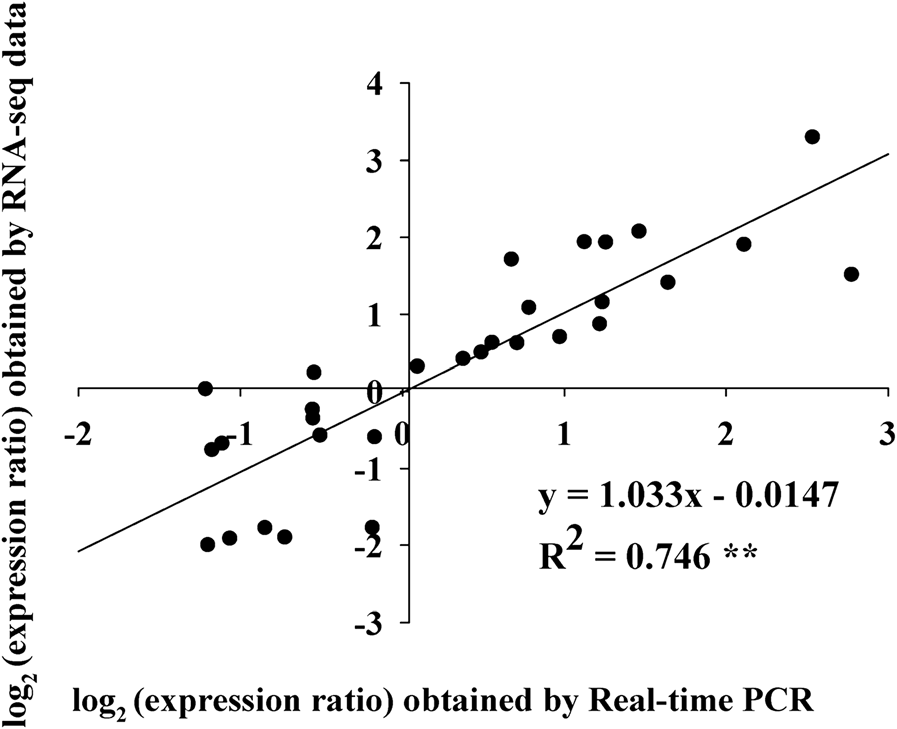
**Coefficient analysis of gene expression levels obtained from RNA-Seq and quantitative real-time PCR data.** The real-time PCR log_2_ values (x-axis) were plotted against coloration stages (y-axis). **indicates a significant difference at p ≤ 0.01.

To verify the results obtained from the RNA-Seq profiling, the expression of fifteen selected genes from the chlorophyll degradation and flavonoid biosynthesis pathways at the three coloration stages were analyzed by real-time PCR in three biological replicates. The real-time PCR data for these genes were generally consistent with the RNA-Seq results. Linear regression [(Q-PCR value) = a (RNA-Seq value) + b] analysis showed an overall correlation coefficient of 0.746** (Figure 10), which indicated that the results of transcriptome analysis were consistent with those of real-time PCR. The real-time PCR value and RPKM value for the fifteen genes are shown in Additional file 11.

### Candidate MYB transcription factors for flavonoid biosynthesis regulation

A phylogenetic tree was constructed using *A. thaliana* MYB TF family members, candidate litchi MYB family members and MYB transcription factors that control anthocyanin, proanthocyanidin and flavonol accumulation in other plant species (Additional file 12). Of the 53 R2R3-MYBs presented in the litchi data set, *Unigene 0017293* was clustered with the TFs known to be involved in regulating anthocyanin biosynthesis. This unigene encodes a protein with 99% amino acid sequence similarity to LcMYB1, which has been reported to control litchi fruit anthocyanin formation [13]. *Unigene 0002564* and *Unigene 0022568* were in the same sub-family as a transcriptional repressor *FaMYB1* [25], while *Unigene 0022702* and *Unigene 0002103* were with *PH4* and *VvMYB5b*, which are genes that potentially regulate anthocyanin and (or) proanthocyanidin biosynthesis [26]. Similarly, *Unigene 0045885*, *Unigene 0050614*, *Unigene 0019166* and *Unigene 0043708* clustered with *AtTT2*, *VvMYBPA1* and *DkMYB2*, candidate genes for proanthocyanidin biosynthesis regulation, and *Unigene 0047595* and *Unigene 0001630* clustered with *AtPFG2*/*AtPFG3*, which regulates flavonol accumulation [27-29]. Other litchi MYB TFs belonging to each of the *A. thaliana* MYB sub-families are also shown in Additional file 12.

## Discussion

### De novo assembled the pericarp transcriptome during coloration

At present no genomic data is available for litchi and only a few hundred cDNA sequences have been published as a public resource [19]; thus more sequence information will be helpful for researches studying this species. Next generation sequencing (NGS) is a fast and effective means to generate transcriptome datasets and high resolution transcriptomic analysis is proving to be of great value for functional genomic study of non-model species [30]. In this current study, a *de novo* transcriptome of the pericarp of litchi was assembled based on short-read (Illumina) sequencing. In total, 51,089 unigenes were assembled with a mean length of 737 bp, which is considerably longer than the previously reported 601 bp for mixed litchi transcriptome [19], 531 bp for Chinese bayberry (*Myrica rubra*) [20] and 508 bp for Chinese white pear (*Pyrus bretschneideri* Rehd) [31]. Approximately 68% of the unigenes were annotated with reference to the nr database, indicating that about one third of the sequences have no apparent homologs, some of which are likely genes with novel functions. In this regard, the transcriptomes of non-model plants, such as Chinese berry (*Myrica rubra*) [20], *Salvia miltiorrhiza* [32], and litchi, will serve as important datasets for studies of taxa specific phenomena.

The RNA-Seq analysis revealed that the numbers and expression profiles of DEGs differed at coloration stages and 3,649 DEGs between the last two developmental stages were identified. Interestingly, this number is similar to that reported for tomato fruit ripening [33]. The amount of information obtained from RNA-Seq analysis was, not surprisingly, much larger than that generated using a suppression subtractive hybridization (SSH) approach, where only 93 unique genes were identified from a litchi floral bud SSH library [34]. We identified 1,800, 1,241, and 2,514 DEGs between green and yellow, yellow and red, and green and red stages, respectively (Figure 4A). These DEGs were clustered into 8 distinct expression patterns, of which cluster 1, 2, and 8 had substantial number of genes, indicating three major gene expression profiles during the pericarp maturation of litchi (Figure 5).

### Candidate genes involved in pericarp maturation of litchi

In this study, 17 TFs were shown to exhibit increasing expression levels during pericarp maturation (Additional file 10) and, of particular note, six NAC TF genes were significantly differentially expressed. NACs comprise a plant-specific TF family with diverse roles in development and stress regulation. For example, NON-RIPENING (NOR), a NAC protein, is necessary for tomato fruit ripening [35]. The *A. thaliana* protein AtNAC078 was found to regulate flavonoid biosynthesis under high-light [36] and we propose that NAC TFs are similarly involved in maturation of the litchi pericarp. Ethylene response factors (ERFs) that regulate apple and kiwifruit fruit ripening have been reported [37,38] and we identified three ERFs in this study that showed differential expressed during litchi pericarp maturation. Although litchi is a non-climacteric fruit, increasing ethylene production has been detected in degreening litchi cultivars and application of the ethylene-releasing chemical ethrel to litchi was effective in enhancing the degradation of chlorophyll in the pericarp [18]. Thus these litchi ERFs may be involved in regulating physiological changes during pericarp maturation. We also identified a range of TFs, including members of the MYB, WD-repeat protein and bHLH families, which yielded no functional annotation after searching NCBI database and these will be the targets of future study.

In addition to TFs, putative signaling factors that are associated with fruit ripening were identified. MAPK signal transduction modules played important roles in regulating many biological processes in plants [39]. In the present study, three genes (*Unigene 0047016*, *Unigene 0023077* and *Unigene 0047066*) encoding putative MAPKs were significantly up-regulated during pericarp maturation. It was reported that an apple MAPK and MAPKKK are involved in abscisic acid (ABA) signal transduction [40] and it was known that ABA plays a crucial role in litchi fruit pigmentation [8,13,18]. Thus we hypothesize that the MAPKs identified in this study may play important roles in the maturation of litchi fruit. Two E3 ubiquitin-protein ligase genes (*Unigene 0050539* and *Unigene 0029707*) were also significantly differentially expressed, indicating the importance of post-transcriptional modification during litchi pericarp maturation. *Unigene 0047192* encodes a GH3 with a high degree of sequence similarity to longan (*Dimocarpus longan*) *DlGH3.1* and *DlGH3.2*, both of which are associated with pericarp growth and fruit maturation [41].

### The pivotal role of SGR in chlorophyll degradation

The degradation of chlorophylls is essential for the strong coloration of fruit crops and a degreening process associated with chlorophyll degradation was observed during litchi coloration (Figure 1). Approximately 80% of the total chlorophyll content was degraded during the pericarp change from the green to the yellow stage. During this period, the expression of putative chlorophyll degradation genes, including two *NYCs,* three *CLHs,* one *HCAR,* two *PAOs* and one *RCCR,* showed a reduction in expression. In contrast, pheophytinase gene (*PPH*) exhibited an increase in transcript levels in the pericarp in parallel with chlorophyll degradation. Recent studies of leaf senescence in *A. thaliana* and rice showed that *PPH* is senescence induced, and that it specifically dephytylates the Mg-free chlorophyll pigment pheophytin (phein), such that the mutant of *PPH* exhibits a stay green phenotype [4]. However, we observed that transient expression of litchi *PPH* in *N. benthamiana* leaf had no obvious effect on chlorophyll degradation (Figure 8A), a result that is consistent with a similar study of a kiwifruit PPH [6].

Stay green (SGR), a chloroplast localized protein, is not a chlorophyll catabolic enzyme, although it regulates chlorophyll degradation and is required for its initiation [4,5]. *Unigene 0048135* was the only *SGR* gene found in the litchi transcriptome database and its expression was observed to increase in parallel with chlorophyll degradation during the developmental transition from the green to the red stage (Figure 7B). This expression pattern was similar to that of *SGR2* from kiwifruit, which was expressed at much higher levels in gold fruit than in green fruit [6]. When the *SGR* genes from kiwifruit were over-expressed in tobacco leaves, degreening was observed [6]. In the present study, when the isolated litchi *SGR* gene was transiently expressed in *N. benthamiana* leaves, degreening and a sharp decrease of fluorescence ratio were observed (Figure 8A-B). These results indicate that this SGR is a key gene in chlorophyll loss in the litchi pericarp during maturation. Recently, a study revealed that SGR regulate lycopene accumulation through direct interaction with a key carotenoid synthetic enzyme SlPSY1 during tomato fruit development [42]. However, its mode of action remains unknown.

### Structural and regulatory genes involved in flavonoid biosynthesis

Anthocyanins, proanthocyanidins and flavonols are the predominant flavonoids in the litchi pericarp (Figure 1) [9]. Genes participating in each step of the flavonoid biosynthesis pathway were found in the transcriptome dataset, indicating that it has high coverage. Many of these responsible appeared to have several family members, implying that one or more rounds of genome duplication may have occurred during litchi genome evolution. The biosynthetic pathway of anthocyanins has been well characterized in fruit species, *such as apple* [12]*, Chinese bayberry* [14]*, and grape* [43]*.* Some genes involved in anthocyanin biosynthesis identified in this study had high similarity to known flavonoid biosynthesis genes, including *CHS* (*Unigene 0020641*), *CHI* (*Unigene 0030141*), *F3H* (*Unigene 0030143*), *ANS* (*Unigene 0004803*) and *UFGT* (*Unigene 0005792*) [8]. While some genes displayed low similarity to known genes suggests that there are new putative genes contributing to this pathway in this transcriptome.

We identified genes encoding enzymes from a broad range of steps in the flavonoid biosynthesis pathway. For example, PAL catalyzes the initial step of the phenylpropanoid pathway, which leads to the synthesis of both lignin and flavonoids. PAL is generally encoded by a small gene family: three *CcPALs* from *Coffea* and five *PtPALs* from *Populus trichocarpa* have been characterized and some family members displayed different expression patterns, suggesting divergent functions [44,45]. Transcripts corresponding to seven *PAL* genes with differential expression patterns during litchi pericarp coloration detected (Figure 9B). Five *4CLs* and two *C4Hs* with differential expression patterns were also identified and higher expression of *CHS* genes and *CHI* genes were observed in the red stage. CHS proteins have multiple functions, such as protection from UV radiation, defense against pathogens, pigment biosynthesis and pollen fertility [46]. Chalcone isomerase-like protein from Japanese morning glory enhances both flavonoid production and flower pigmentation, while tomato *SlCHI1* was found to have a key role in terpenoid production [47]. The increased expressions of *CHS*, *CHI* and *F3H* genes do not correlate exactly with the decreased concentration of total flavonoids during pericarp coloration. This was probably due to the complicate composition of flavonoids. In the pericarp of litchi, the decrease of total flavonoids mainly result from the drop of proanthocyanins during coloration (Figure 1). Further study is needed to clarify the functions of these genes form litchi.

*DFR*, *ANS* and *UFGT* are late anthocyanin biosynthetic genes, which are coordinately expressed during red coloration in apple skin, where their levels of expression positively correlate with anthocyanin concentration [12]. In this study, other than the *UFGT* genes *Unigene 0016938* and *Unigene 001639*, these late structural genes showed the highest expression levels in the pericarp with the highest anthocyanin concentration (Figure 9B). Similar to the situation with mangosteen, UFGT is considered to be a key enzyme in litchi anthocyanin biosynthesis [8,48]. Of the five *UFGT* genes, the expression levels of *Unigene 0005792*, *Unigene 0040379* and *Unigene 0040034* increased in parallel with the accumulation of anthocyanins during fruit coloration. In *Vitis vinifera*, seven *UFGT* genes contribute to the chemical diversity of bioactive flavonol glycosides [49]. In the present study, the expressions of *Unigene 0016938* and *Unigene 001639* were not paralleled with the accumulation of anthocyanins. As mentioned earlier, these two unigenes showed high identity with UDP-galactose: flavonoid glycosyltransferase in pear [24]. These results suggested that *Unigene 0016938* and *Unigene 0016939* might be other glycosyltransferases for other flavonoids.

Glutathione *S*-transferase (GST) is necessary for the transport of anthocyanins from the cytosol to the vacuole and the *A. thaliana GST* mutant *tt19* accumulates extremely low levels of anthocyanins [50,51]. GSTs that are responsible for anthocyanin transport have also been identified in grape berry [52]. In the present study, a *GST* gene (*Unigene 0021409*) was found significantly up-regulated during litchi pericarp coloration.

The synthesis of proanthocyanidins and flavonols share common biosynthesis steps with anthocyanin. Anthocyanidin reductase (ANR) and leucoanthocyanidin reductase (LAR) play important roles in the biosynthesis of proanthocyanidins, while flavonol synthase (FLS) is specifically involved in the biosynthesis the flavonols catechin and epicatechin [53,54]. Indeed, a close relationship between anthocyanin and proanthocyanidin accumulation has been noticed. Over-expression of *ANR* and *LAR* in tobacco results in both a decrease in anthocyanin levels and an increase in the concentration of proanthocyanidins in flower [53,55]. As shown in Figure 1, proanthocyanidin levels decreased coincident with an increase in anthocyanin abundance during litchi pericarp coloration. However, two *ANR* genes and one of the two *LAR* genes showed the highest expression in red pericarp, the stage with the lowest proanthocyanidin concentration.

In plants, MYB TFs typically comprise large families which collectively function in a wide variety of plant-specific processes, as has been shown by extensive functional characterization in *A. thaliana* [56]. In the present study, 53 litchi R2R3-MYB TFs were identified as being expressed in the fruit pericarp. As has been reported for many other fruits, R2R3-MYB TFs and anthocyanin biosynthetic genes are coordinately expressed during ripening coincident with anthocyanin accumulation [14,15,57,58]. *LcMYB1* has been reported to control anthocyanin biosynthesis in litchi [13] and MYB TFs have also been shown to regulate the biosynthesis of flavonol and proanthocyanidins in *A. thaliana* and gentian flowers [29,59]. Moreover, TT2, a MYB TF, activates *BAN* (*ANR*) expression and enhances proanthocyanidin biosynthesis in seed coat of *A. thaliana* [28], while *VvMYBPA2*, *VvMYBPA1* and *VvMYB5b* were shown to regulate anthocyanin and (or) proanthocyanidin biosynthesis in developing grape berries [26,27,60]. However, strawberry *FaMYB1* was reported to suppress anthocyanin and flavonol accumulation in transgenic tobacco lines and over-expression of this gene inhibited the biosynthesis of proanthocyanidins in the leaves of *Lotus corniculatus* [25,61]. These results suggested the multiple and complex functions of MYB TFs. In this current study, 11 litchi MYB TFs were shown to align within the same sub-group with gene known to be involved in the regulation of flavonoid biosynthesis in other plant species (Additional file 12), from which we infer that those genes may have similar functions in regulating the accumulation of flavonoids in the litchi pericarp. These genes will the target of future researches into the regulation of flavonoid biosynthesis in litchi.

## Conclusions

The pericarp of litchi has been the focus of studies associated with fruit size, coloration, cracking and shelf life. In this study, an analysis of the transcriptome of litchi pericarp was performed through de novo assembly of next generation sequencing data to obtain information regarding the molecular mechanisms underlying the physiological changes in the pericarp, including those leading to fruit surface coloration. A total of 4.7 Gb of raw RNA-Seq data was generated and this was then de novo assembled into 51,089 unigenes with a mean length of 737 bp. This study provides a large collection of transcripts and expression profiles associated with litchi fruit maturation processes. Genes encoding enzymes involved in chlorophyll degradation and flavonoid biosynthesis were identified in the transcriptome dataset. The results demonstrated that Stay Green (SGR) protein might play a key role in chlorophyll degradation in the litchi pericarp. The expression levels of most genes especially late anthocyanin biosynthesis genes were found co-ordinated up-regulated coincident with the accumulation of anthocyanins. The candidate MYB transcription factors that likely regulate flavonoid biosynthesis were also identified. Their possible roles in anthocyanin biosynthesis were discussed. This study provides a platform for litchi functional genomic research within this species and will benefit researches in other closely related species.

## Methods

### Plant materials

Litchi trees of the cultivar Nuomici, which exhibits clear degreening and coloration in fruit skin during maturation, were grown in an experimental orchard at South China Agricultural University (Guangzhou, China). Trees used for sample collection were cultivated using standard horticultural practices and methods for disease and insect control. Fruit pericarp material was collected at three different developmental stages on fruit coloration: 52 days after anthesis (DAA; Green stage), 62 DAA (Yellow stage) and 72 DAA (Red stage), as shown in Figure 1. Three fruits from each plant and each stage were collected and immediately frozen in liquid nitrogen and stored at −80°C until use.

### Flavonoid and chlorophyll measurements

Total anthocyanin levels were determined as described by Wei et al. [8], by measuring the absorbance at 520 nm of samples extracted and diluted with pH 1.0 and 4.5 buffers. Proanthocyanidin and flavonoid levels were measured as previously described [9] and chlorophyll levels were measured according to the protocol of Arnon [62].

### RNA extraction, library construction and RNA-Sequencing

Transcriptome libraries were generated by pooling equal quantities of RNA from pericarp from each of the three fruit developmental stages, while the three DGE libraries consisted of separate RNA extracts from pericarp from each of the three fruit developmental stages. Each of these libraries consisted of equal amounts of RNA from three biological replicates of each developmental stage. Total RNA was extracted using the RNA_OUT_ kit (Tiandz, Beijing, China). DNase I (TaKaRa, Japan) was added to remove genomic DNA and then RNA integrity was confirmed using a 2100 Bioanalyzer (Agilent Technologies, Santa Clara, CA, USA). Since RNA-Seq data is typically highly reproducible with relatively little technical variation, each DGE library was only sequenced once [21]. The steps of mRNA enrichment, mRNA fragmentation, second-strand cDNA synthesis, size selection, PCR amplification and subsequent sequencing using an Illumina HiSeq™ 2000 (San Diego, CA, USA) were performed by the Beijing Genome Institute (BGI) (Shenzhen, China).

### De novo assembly and functional annotation

Prior to bioinformatic analysis, the raw sequences were filtered to remove reads with only adaptor sequence, reads with more than 5% unknown nucleotides, and low-quality reads with more than 20% bases with a quality value ≤10. *De novo* assembly was carried out by the BGI using the short-read assembly program Trinity with the following parameters: min_contig_length = 200; min_glue = 2; group_pairs_distance = 200; path_reinforcement_distance = 75; bfly_opts = ‘-V 10; −edge-thr = 0.05; and –stderr’, min_kmer_cov = 1 [63]. A total of 96,771 transcripts assembled by Trinity were collapsed into 57,050 transcripts and redundant unigenes were then removed using TGICL (v.2.1) with the options ‘-l 100 -v 30’ [64].

Functional annotation of the unigenes was performed using the NCBI non-redundant (nr) database (http://www.ncbi.nlm.nih.gov), the Swiss-Prot protein database (http://www.expasy.ch/sprot), the Kyoto Encyclopedia of Genes and Genomes (KEGG) database (http://www.genome.jp/kegg) [65], and the Clusters of Orthologous Groups of proteins (COG) database (http://www.ncbi.nlm.nih.gov/COG) using BLASTx with an E-value <10^−5^. The best matched hits were used to determine the sequence direction of each unigene. If the results from the different databases conflicted with each other, a priority order of nr, Swiss-Prot, KEGG and COG should be followed when deciding the sequence direction of unigenes. When a unigene did not align to any of the above databases, the ESTScan software was used to establish the sequence direction [66]. The 25 top-hit species were identified using nr annotation and gene ontology (GO) was assigned by WEGO using the GO IDs annotated by the Blast2GO software (Version 2.3.4) [67]. The KEGG pathway annotation was performed using the BLAST software against the KEGG database, while COG was used to match each annotated unigene to an ancient conserved domain, and represented major phylogenetic lineages of litchi.

### Read mapping and quantification of gene expression

In order to obtain uncontaminated sequences, reads containing adaptors, reads with more than 10% unknown nucleotides, and low-quality reads with more than 50% bases with a quality value ≤5 were removed. A sequence alignment package, SOAPaligner (Version 2.20) was then used to map reads to the transcriptome using the parameters: “-m 0 -× 1000 -s 40 -l 35 -v 3 -r 2”. Details of the parameters are described at the following web address: http://soap.genomics.org.cn/soapaligner.html. The number of mapped and filtered reads for each unigene was calculated and normalized giving the corresponding RPKM (Reads Per kb per Million reads) values [68]. As previously described [20], the differentially expressed unigenes between two samples were determined based on a false discovery rate (FDR) threshold of < 0.001, an absolute log2 fold change value of >1.0 and a P value <0.01.

### Quantitative real-time PCR analysis

Total RNA from the pericarp of litchi fruits was extracted as described above and cDNA was synthesized from total RNA (2 μg) using oligo (dT) primers and M-MLV reverse transcriptase, according to the manufacturer’s instructions (Invitrogen, USA) in a 20 μl total volume. The specific real-time PCR primers were designed using the BatchPrimer3 program [69] (Additional file 13). Transcript levels were analyzed by quantitative real-time PCR using the THUNDERBIRD Real-Time PCR Mix (TOYOBO, Japan) and an ABI 7500 Real-Time PCR System (Applied Biosystems, USA) according to the manufacturers’ instructions [13]. All biological replicates were analyzed in triplicate. Real-time PCR reactions were normalized to the Ct values for litchi *LcActin* (HQ615689) and *LcGAPDH* (JF759907). The relative expression levels of the target genes were calculated using the formula 2^-△△CT^.

### Phylogenetic analysis

Phylogenetic analysis based on amino acid sequences were performed using MEGA (version 5.02) and the Neighbor-joining method with 1,000 bootstrap replicates [70].

### Functional characterization of *LcSGR* by transient transformation in *Nicotiana benthamiana*

The plasmids used in the transient expression assay were constructed by ligating full-length *LcSGR* (GenBank: KP853137) and *LcPPH* (GenBank: KP853136) into the pEAQ-HT vector [71]. The primers used to amplify the coding regions were: LcSGR-F: TTCTGCCCAAATTCGCGAATGGGTACTTTGGTTGCTG and LcSGR-R: AGTTAAAGGCCTCGAGTCAAATTTGTTGTTGCAAGCTC, LcPPH-F: TTCTGCCCAAATTCGCGAATGGAAATTATCTCATATAATTGCC and LcPPH-R: AGTTAAAGGCCTCGAGTCAAGAAGATTTAACTTCTAATTTCC. The products were recombined with the linearized vector pEAQ-HT by restriction enzyme sites *Nru* I and *Xho* I (In-Fusion™ Advantage PCR Cloning Kits; Clontech). The resulting vectors (pEAQ-LcSGR or pEAQ-LcPPH) were introduced into *Agrobacterium tumefaciens* strain GV3101.

*N. benthamiana* plants were grown under standard greenhouse conditions at 22°C using natural light with 16 h daylight and 8 h dark. Plants with a minimum of six leaves were used for infiltration and kept in the greenhouse for the duration of the experiment. *A. tumefaciens* cultures harboring pEAQ-LcSGR or pEAQ-LcPPH were infiltrated into *N. benthamiana* leaves, as described in Sainsbury et al. [71]. Controls were infiltrated with an empty pEAQ-HT construct in parallel. Leaves were photographed 4 days after infiltration and chlorophyll fluorescence kinetic parameters were measured with an FMS-2 (Hansatech, Norfolk, UK) modulated chlorophyll fluorometer.

### Availability of supporting data

The assembled transcriptomic sequences have been deposited at DDBJ/EMBL/GenBank under the accession number GenBank: GBRI01000000 (http://www.ncbi.nlm.nih.gov/genbank). The raw data for the DGE analysis were also deposited in the NCBI Sequence Read Archive under accession numbers SRX700596 (Green stage), SRX700598 (yellow stage), and SRX700599 (Red stage) (http://www.ncbi.nlm.nih.gov/sra/). Accession numbers of protein sequences used for phylogenetic analysis in this article can be found in Additional file 12.

## Additional files

Additional file 1:
**Length distribution of contigs, unigenes, CDS and proteins from the assembled transcriptome of litchi.**
Additional file 2:
**KEGG pathways involved in litchi fruit development.**
Additional file 3:
**COG classification of litchi unigenes.**
Additional file 4:
**Distribution of reads on reference genes.** Three libraries showed good levels of randomness, with the number of reads evenly distributed over reference genes. A, B and C represent green, yellow and red libraries, respectively. Additional file 5:
**Percentage of unigenes mapped in the litchi transcriptome data that were expressed at three developmental stages.** Gene coverage is the percentage of a gene for which there are matching reads. This value is the ratio of the number of bases in a gene that correspond to unique mapping reads to the total number of bases in that gene. The distribution of unique reads over different read abundance categories show similar patterns for all three RNA-Seq libraries. A, B and C represent green, yellow and red libraries, respectively. Additional file 6:
**Differentially-expressed genes between the yellow and green libraries.** RPKM, Reads Per Kb per Million reads; P-value, p value for hypothesis testing; and FDR: false discovery rate. We used FDR ≤ 0.001 and the absolute value of log2 Ratio ≥ 1 as the thresholds to judge the significance of gene expression differences. Additional file 7:
**Differentially expressed genes (DEGs) between the red and yellow libraries.** RPKM, Reads Per Kb per Million reads; P-value, p value for hypothesis testing; and FDR: false discovery rate. We used FDR ≤ 0.001 and the absolute value of log2 Ratio ≥ 1 as the thresholds to judge the significance of gene expression differences. Additional file 8:
**Differentially-expressed genes between the red and green libraries.** RPKM: Reads Per Kb per Million reads. P-value: pvalue for hypothesis testing. FDR: false discovery rate. We used FDR ≤ 0.001 and the absolute value of log2 Ratio ≥ 1 as the threshold to judge the significance of gene expression difference. Additional file 9:
**Histogram representation of the functional categories distribution is expressed as percentage of the amount of genes belonging to the cluster.**
Additional file 10:
**156 genes differentially expressed during maturation.** 156 genes were found that were significantly differentially-expressed genes between green and yellow, between yellow and red, and between green and red. Annotations and their expression levels for the 156 genes were showed in this table. Additional file 11:
**The real-time PCR value and RPKM value for the selected fifteen genes.**
Additional file 12:
**Schematic representation of the relationships between different R2R3-MYB subgroups.** The tree was inferred using the Neighbor-joining method and 1,000 bootstraps with putative amino acid MYB sequences with MEGA 5.0 software. The numbers before the *A. thaliana* gene ID are subgroup numbers and were designated as previously reported [48]. GenBank protein accession numbers for anthocyanin related MYB transcription factors are listed in Lai et al. [13]. Additional GenBank accession numbers for each gene are as follows: LcSGR, GenBank: KP853137; AtSGR1, GenBank: NP_567673.1; AtSGR2, GenBank: NP_192928.2; AtSGR3, GenBank: NP_564489.1; CaSGR, GenBank: ABX82698.1; GmSGR1, GenBank: NP_001238357.1; GmSGR2, GenBank: NP_001236690.1; HvSGR, GenBank: AAW82955.1; NtSGR, GenBank: ABY19382.1; OsSGR1, GenBank: NP_001063758.1; OsSGR2, GenBank: NP_001054370.1; OsSGR3, GenBank: CAE05787.3; PsSGR, GenBank: A7VLV1.1; SlSGR, GenBank: NP_001234723.1; SbSGR, GenBank: AAW82958.1; ZmSGR1, GenBank: NP_001105770.1; ZmSGR2, GenBank: NP_001105771.1; AdSGR1, GenBank: FG509383; VvMYBPA1, GenBank: NM_001281231.1; DkMyb4, GenBank: AB503701.1; LjTT2c, GenBank: AB300035.1; LjTT2a, GenBank: AB300033.2; LjTT2c, GenBank: AB300034.2; DkMyb2, GenBank: AB503699.1; VvMybPA2, GenBank: NM_001281024.1; MtPAR, GenBank: HQ337434.1; TaMYB14-1, GenBank: JN049641.1; GtMYBP3, GenBank: AB733016.1; GtMYBP4, GenBank: AB289446.1. VvMYBF1, GenBank: FJ948477.2; and SbMYBY1, GenBank: AY860968.1. *Arabidopsis thaliana* R2R3-MYB family genes were downloaded from the ‘The Database of *Arabidopsis* Transcription Factors’ (http://datf.cbi.pku.edu.cn/). Additional file 13:
**Primers used in real-time PCR analysis to validate expression levels of 15 genes during litchi fruit development.**

## Abbreviations

4CL: 4 coumarate CoA ligase
ANR: Anthocyanidin reductase
ANS/LDOX: Anthocyanidin synthase/leucoanthocyanidin dioxygenase
C4H: Cinnamic acid 4-hydroxylase
CHI: Chalcone isomerase
Chl: Chlorophyll
CHS: Chalcone synthase
CLH: Chlorophyllase
COG: Cluster of orthologous groups of proteins
DEG: Differentially expressed gene
DFR: Dihydroflavonol reductase
DGE: Digital gene expression
EST: Expressed sequence tag
F3H: Flavanone 3-hydroxylase
F3′H: Flavanone 3-hydroxylase
FLS: Flavonol synthase
GH3: Gretchenhagen-3
GO: Gene ontology
GST: Glutathione *S*-transferase
HCAR: Hydroxy-Chl a reductase
KEGG: Kyoto encyclopedia of genes and genomes
LAR: Leucoanthocyanidin reductase
MAPK: Mitogen activated protein kinase
MAPKKK: Mitogen activated protein kinase kinase kinase
MCS: Metal chelating substance
NCCs: Nonfluorescent Chl catabolites
NOL: NYC1-like
NYC1: Non-yellow coloring 1
PAL: Phenylalanine ammonia lyase
PAO: Pheide a oxygenase
pFCC: Primary fluorescent Chl catabolite
Pheide: Pheophorbide
PPH: Pheophytinase
RCC: Red Chl catabolite
RCCR: RCC reductase
SGR: Stay-green protein
UFGT: UDP-flavonoid glucosyltransferase

## References

[1] Andersen Ø, Jordheim M, Andersen ØM, Markham KR (2006). The anthocyanins. Flavonoids.

[2] Wang HC, Huang XM, Hu GB, Yang Z, Huang HB (2005). A comparative study of chlorophyll loss and its related mechanism during fruit maturation in the pericarp of fast- and slow-degreening litchi pericarp. Sci Hortic.

[3] Jose AM, Schafer E (1978). Distorted phytochrome action spectra in green plants. Planta.

[4] Hörtensteiner S (2013). Update on the biochemistry of chlorophyll breakdown. Plant Mol Biol.

[5] Sakuraba Y, Schelbert S, Park SY, Han SH, Lee BD, Andres CB (2012). STAY-GREEN and chlorophyll catabolic enzymes interact at light-harvesting complex II for chlorophyll detoxification during leaf senescence in *Arabidopsis*. Plant Cell.

[6] Pilkington SM, Montefiori M, Jameson PE, Allan AC (2012). The control of chlorophyll levels in maturing kiwifruit. Planta.

[7] Winkel BS, Grotewold E (2006). The biosynthesis of flavonoids. The science of flavonoids.

[8] Wei YZ, Hu FC, Hu GB, Li XJ, Huang XM, Wang HC (2011). Differential expression of anthocyanin biosynthetic genes in relation to anthocyanin accumulation in the pericarp of *Litchi chinensis* Sonn. PLoS One.

[9] Wang H, Hu Z, Wang Y, Chen H, Huang X (2011). Phenolic compounds and the antioxidant activities in litchi pericarp: Difference among cultivars. Sci Hortic.

[10] Ogasawara J, Kitadate K, Nishioka H, Fujii H, Sakurai T, Kizaki T (2009). Oligonol, a new lychee fruit-derived low-molecular form of polyphenol, enhances lipolysis in primary rat adipocytes through activation of the ERK1/2 pathway. Phytother Res.

[11] Jiang Y, Duan X, Joyce D, Zhang Z, Li J (2004). Advances in understanding of enzymatic browning in harvested litchi fruit. Food Chem.

[12] Honda C, Kotoda N, Wada M, Kondo S, Kobayashi S, Soejima J (2002). Anthocyanin biosynthetic genes are coordinately expressed during red coloration in apple skin. Plant Physiol Bioch.

[13] Lai B, Li XJ, Hu B, Qin YH, Huang XM, Wang HC (2014). LcMYB1 is a key determinant of differential anthocyanin accumulation among genotypes, tissues, developmental phases and ABA and light stimuli in *Litchi chinensis*. PLoS One.

[14] Niu SS, Xu CJ, Zhang WS, Zhang B, Li X, Lin-Wang K (2010). Coordinated regulation of anthocyanin biosynthesis in Chinese bayberry (*Myrica rubra*) fruit by a R2R3 MYB transcription factor. Planta.

[15] Chagne D, Lin-Wang K, Espley RV, Volz RK, How NM, Rouse S (2013). An ancient duplication of apple MYB transcription factors is responsible for novel red fruit-flesh phenotypes. Plant Physiol.

[16] Hichri I, Barrieu F, Bogs J, Kappel C, Delrot S, Lauvergeat V (2011). Recent advances in the transcriptional regulation of the flavonoid biosynthetic pathway. J Exp Bot.

[17] Underhill S, Critchley C (1992). The physiology and anatomy of lychee (*Litchi chinensis* Sonn.) pericarp during fruit development. J Hort Sci.

[18] Wang H, Huang H, Huang X (2007). Differential effects of abscisic acid and ethylene on the fruit maturation of *Litchi chinensis* Sonn. Plant Growth Regul.

[19] Li C, Wang Y, Huang X, Li J, Wang H, Li J (2013). De novo assembly and characterization of fruit transcriptome in *Litchi chinensis* Sonn and analysis of differentially regulated genes in fruit in response to shading. BMC Genomics.

[20] Feng C, Chen M, Xu CJ, Bai L, Yin XR, Li X (2012). Transcriptomic analysis of Chinese bayberry (*Myrica rubra*) fruit development and ripening using RNA-Seq. BMC Genomics.

[21] Liu G, Li W, Zheng P, Xu T, Chen L, Liu D (2012). Transcriptomic analysis of ‘Suli’ pear (*Pyrus pyrifolia* white pear group) buds during the dormancy by RNA-Seq. BMC Genomics.

[22] Tatusov RL, Galperin MY, Natale DA, Koonin EV (2000). The COG database: a tool for genome-scale analysis of protein functions and evolution. Nucleic Acids Res.

[23] Deroles S, Kevin G, Kevin D, Chris W (2009). **Anthocyanin biosynthesis in plant cell cultures: A potential source of natural colourants**. Anthocyanins: Biosynthesis, functions and applications. pp 107–117.

[24] Zhang D, Yu B, Bai J, Qian M, Shu Q, Su J (2012). Effects of high temperatures on UV-B/visible irradiation induced postharvest anthocyanin accumulation in ‘Yunhongli No. 1’ (*Pyrus pyrifolia* Nakai) pears. Sci Hortic.

[25] Aharoni A, De Vos CH, Wein M, Sun Z, Greco R, Kroon A (2001). The strawberry *FaMYB1* transcription factor suppresses anthocyanin and flavonol accumulation in transgenic tobacco. Plant J.

[26] Deluc L, Bogs J, Walker AR, Ferrier T, Decendit A, Merillon JM (2008). The transcription factor VvMYB5b contributes to the regulation of anthocyanin and proanthocyanidin biosynthesis in developing grape berries. Plant Physiol.

[27] Bogs J, Jaffe FW, Takos AM, Walker AR, Robinson SP (2007). The grapevine transcription factor *VvMYBPA1* regulates proanthocyanidin synthesis during fruit development. Plant Physiol.

[28] Nesi N, Jond C, Debeaujon I, Caboche M, Lepiniec L (2001). The *Arabidopsis TT2* gene encodes an R2R3 MYB domain protein that acts as a key determinant for proanthocyanidin accumulation in developing seed. Plant Cell.

[29] Stracke R, Jahns O, Keck M, Tohge T, Niehaus K, Fernie AR (2010). Analysis of PRODUCTION OF FLAVONOL GLYCOSIDES-dependent flavonol glycoside accumulation in *Arabidopsis thaliana* plants reveals MYB11-, MYB12- and MYB111-independent flavonol glycoside accumulation. New Phytol.

[30] Mutz KO, Heilkenbrinker A, Lonne M, Walter JG, Stahl F (2013). Transcriptome analysis using next-generation sequencing. Curr Opin Biotechnol.

[31] Xie M, Huang Y, Zhang Y, Wang X, Yang H, Yu O (2013). Transcriptome profiling of fruit development and maturation in Chinese white pear (*Pyrus bretschneideri* Rehd). BMC Genomics.

[32] Hua WP, Zhang Y, Song J, Zhao LJ, Wang ZZ (2011). *De novo* transcriptome sequencing in *Salvia miltiorrhiza* to identify genes involved in the biosynthesis of active ingredients. Genomics.

[33] Alba R, Payton P, Fei Z, McQuinn R, Debbie P, Martin GB (2005). Transcriptome and selected metabolite analyses reveal multiple points of ethylene control during tomato fruit development. Plant Cell.

[34] Liu WW, Kim HJ, Chen HB, Lu XY, Zhou BY (2013). Identification of MV-generated ROS responsive EST clones in floral buds of *Litchi chinensis* Sonn. Plant Cell Rep.

[35] Martel C, Vrebalov J, Tafelmeyer P, Giovannoni JJ (2011). The tomato MADS-box transcription factor RIPENING INHIBITOR interacts with promoters involved in numerous ripening processes in a COLORLESS NONRIPENING-dependent manner. Plant Physiol.

[36] Morishita T, Kojima Y, Maruta T, Nishizawa-Yokoi A, Yabuta Y, Shigeoka S (2009). *Arabidopsis* NAC transcription factor, ANAC078, regulates flavonoid biosynthesis under high-light. Plant Cell Physiol.

[37] Yin XR, Allan AC, Chen KS, Ferguson IB (2010). Kiwifruit *EIL* and *ERF* genes involved in regulating fruit ripening. Plant Physiol.

[38] Tacken E, Ireland H, Gunaseelan K, Karunairetnam S, Wang D, Schultz K (2010). The role of ethylene and cold temperature in the regulation of the apple *POLYGALACTURONASE1* gene and fruit softening. Plant Physiol.

[39] Hamel LP, Nicole MC, Sritubtim S, Morency MJ, Ellis M, Ehlting J (2006). Ancient signals: comparative genomics of plant *MAPK* and *MAPKK* gene families. Trends Plant Sci.

[40] Wang XJ, Zhu SY, Lu YF, Zhao R, Xin Q, Wang XF (2010). Two coupled components of the mitogen-activated protein kinase cascade MdMPK1 and MdMKK1 from apple function in ABA signal transduction. Plant Cell Physiol.

[41] Kuang JF, Zhang Y, Chen JY, Chen QJ, Jiang YM, Lin HT (2011). Two *GH3* genes from longan are differentially regulated during fruit growth and development. Gene.

[42] Luo Z, Zhang J, Li J, Yang C, Wang T, Ouyang B (2013). A STAY-GREEN protein SlSGR1 regulates lycopene and beta-carotene accumulation by interacting directly with SlPSY1 during ripening processes in tomato. New Phytol.

[43] Boss PK, Davies C, Robinson SP (1996). Analysis of the expression of anthocyanin pathway genes in developing Vitis vinifera L. cv Shiraz grape berries and the implications for pathway regulation. Plant Physiol.

[44] Lepelley M, Mahesh V, McCarthy J, Rigoreau M, Crouzillat D, Chabrillange N (2012). Characterization, high-resolution mapping and differential expression of three homologous *PAL* genes in *Coffea canephora* Pierre (Rubiaceae). Planta.

[45] Shi R, Shuford CM, Wang JP, Sun YH, Yang Z, Chen HC (2013). Regulation of phenylalanine ammonia-lyase (*PAL*) gene family in wood forming tissue of *Populus trichocarpa*. Planta.

[46] Deng X, Bashandy H, Ainasoja M, Kontturi J, Pietiainen M, Laitinen RA (2014). Functional diversification of duplicated *chalcone synthase* genes in anthocyanin biosynthesis of *Gerbera hybrida*. New Phytol.

[47] Kang JH, McRoberts J, Shi F, Moreno JE, Jones AD, Howe GA (2014). The flavonoid biosynthetic enzyme chalcone isomerase modulates terpenoid production in glandular trichomes of tomato. Plant Physiol.

[48] Palapol Y, Ketsa S, Lin-Wang K, Ferguson IB, Allan AC (2009). A MYB transcription factor regulates anthocyanin biosynthesis in mangosteen (*Garcinia mangostana* L.) fruit during ripening. Planta.

[49] Ono E, Homma Y, Horikawa M, Kunikane-Doi S, Imai H, Takahashi S (2010). Functional differentiation of the glycosyltransferases that contribute to the chemical diversity of bioactive flavonol glycosides in grapevines (*Vitis vinifera*). Plant Cell.

[50] Marrs KA, Alfenito MR, Lloyd AM, Walbot V (1995). A glutathione *S*-transferase involved in vacuolar transfer encoded by the maize gene *Bronze-2*. Nature.

[51] Sun Y, Li H, Huang JR (2012). *Arabidopsis* TT19 functions as a carrier to transport anthocyanin from the cytosol to tonoplasts. Mol Plant.

[52] Conn S, Curtin C, Bezier A, Franco C, Zhang W (2008). Purification, molecular cloning, and characterization of glutathione *S*-transferases (GSTs) from pigmented *Vitis vinifera* L. cell suspension cultures as putative anthocyanin transport proteins. J Exp Bot.

[53] Xie DY, Sharma SB, Paiva NL, Ferreira D, Dixon RA (2003). Role of anthocyanidin reductase, encoded by *BANYULS* in plant flavonoid biosynthesis. Science.

[54] Bogs J, Downey MO, Harvey JS, Ashton AR, Tanner GJ, Robinson SP (2005). Proanthocyanidin synthesis and expression of genes encoding leucoanthocyanidin reductase and anthocyanidin reductase in developing grape berries and grapevine leaves. Plant Physiol.

[55] Liu Y, Shi Z, Maximova S, Payne MJ, Guiltinan MJ (2013). Proanthocyanidin synthesis in *Theobroma cacao*: genes encoding anthocyanidin synthase, anthocyanidin reductase, and leucoanthocyanidin reductase. BMC Plant Biol.

[56] Dubos C, Stracke R, Grotewold E, Weisshaar B, Martin C, Lepiniec L (2010). MYB transcription factors in *Arabidopsis*. Trends Plant Sci.

[57] Umemura H, Otagaki S, Wada M, Kondo S, Matsumoto S (2013). Expression and functional analysis of a novel *MYB* gene, *MdMYB110a_JP*, responsible for red flesh, not skin color in apple fruit. Planta.

[58] Feng S, Wang Y, Yang S, Xu Y, Chen X (2010). Anthocyanin biosynthesis in pears is regulated by a R2R3-MYB transcription factor PyMYB10. Planta.

[59] Nakatsuka T, Saito M, Yamada E, Fujita K, Kakizaki Y, Nishihara M (2012). Isolation and characterization of GtMYBP3 and GtMYBP4, orthologues of R2R3-MYB transcription factors that regulate early flavonoid biosynthesis, in gentian flowers. J Exp Bot.

[60] Terrier N, Torregrosa L, Ageorges A, Vialet S, Verries C, Cheynier V (2009). Ectopic expression of VvMybPA2 promotes proanthocyanidin biosynthesis in grapevine and suggests additional targets in the pathway. Plant Physiol.

[61] Paolocci F, Robbins MP, Passeri V, Hauck B, Morris P, Rubini A (2011). The strawberry transcription factor *FaMYB1* inhibits the biosynthesis of proanthocyanidins in *Lotus corniculatus* leaves. J Exp Bot.

[62] Arnon DI (1949). Copper enzymes in isolated chlroplasts. Polyphenoxidase in *Beta vulgaris*. Plant Physiol.

[63] Grabherr MG, Haas BJ, Yassour M, Levin JZ, Thompson DA, Amit I (2011). Full-length transcriptome assembly from RNA-Seq data without a reference genome. Nat Biotechnol.

[64] Pertea G, Huang X, Liang F, Antonescu V, Sultana R, Karamycheva S (2003). TIGR Gene Indices clustering tools (TGICL): a software system for fast clustering of large EST datasets. Bioinformatics.

[65] Kanehisa M, Araki M, Goto S, Hattori M, Hirakawa M, Itoh M (2008). KEGG for linking genomes to life and the environment. Nucleic Acids Res.

[66] Iseli C, Jongeneel CV, Bucher P. ESTScan: a program for detecting, evaluating, and reconstructing potential coding regions in EST sequences. Proc Int Conf Intell Syst Mol Biol. 1999:138-48. 10786296

[67] Gotz S, Garcia-Gomez JM, Terol J, Williams TD, Nagaraj SH, Nueda MJ (2008). High-throughput functional annotation and data mining with the Blast2GO suite. Nucleic Acids Res.

[68] Mortazavi A, Williams BA, McCue K, Schaeffer L, Wold B (2008). Mapping and quantifying mammalian transcriptomes by RNA-Seq. Nat Methods.

[69] You FM, Huo N, Gu YQ, Luo MC, Ma Y, Hane D (2008). BatchPrimer3: a high throughput web application for PCR and sequencing primer design. BMC Bioinformatics.

[70] Tamura K, Peterson D, Peterson N, Stecher G, Nei M, Kumar S (2011). MEGA5: molecular evolutionary genetics analysis using maximum likelihood, evolutionary distance, and maximum parsimony methods. Mol Biol Evol.

[71] Sainsbury F, Thuenemann EC, Lomonossoff GP (2009). pEAQ: versatile expression vectors for easy and quick transient expression of heterologous proteins in plants. Plant Biotechnol J.

