# Supplementary figures and images for: Transcriptomic analysis of Litchi chinensis pericarp during maturation with a focus on chlorophyll degradation and flavonoid biosynthesis
Source: BMC Genomics. 2015 Mar 21;16(1):225. doi: 10.1186/s12864-015-1433-4 (PMC4376514; doi:10.1186/s12864-015-1433-4)

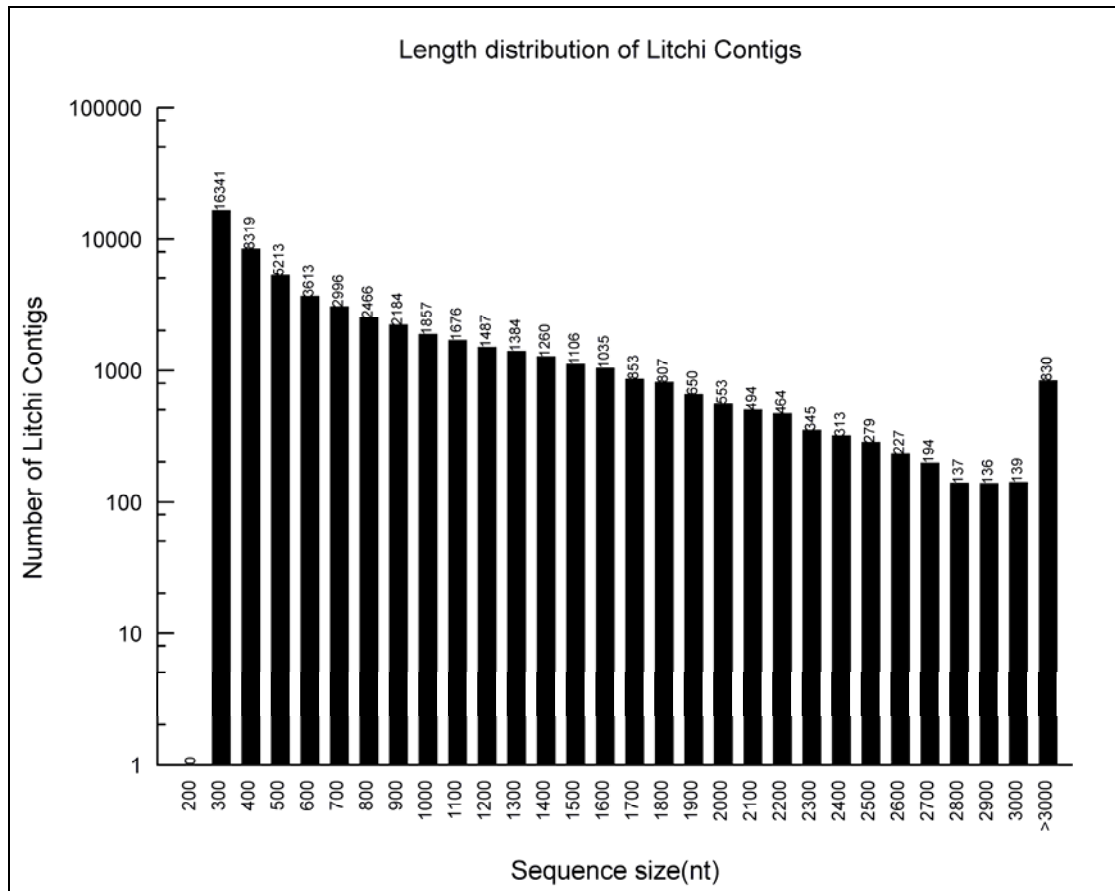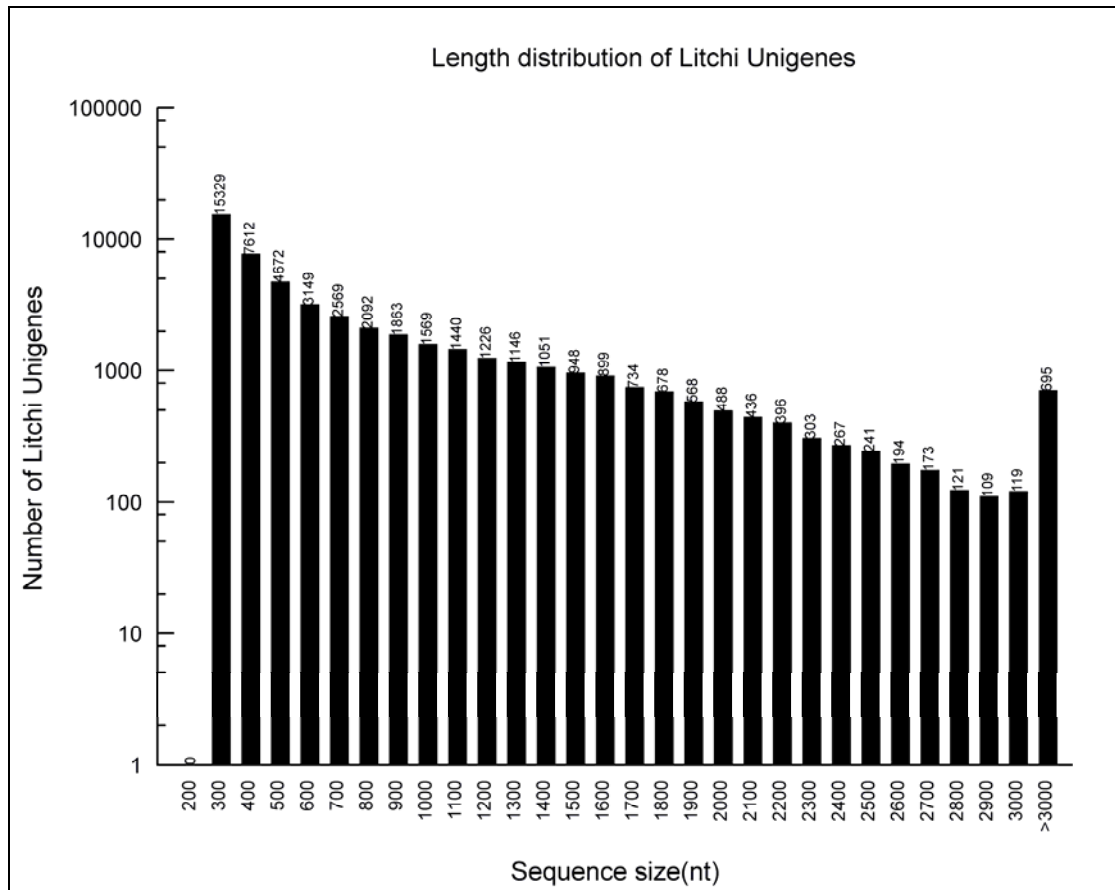

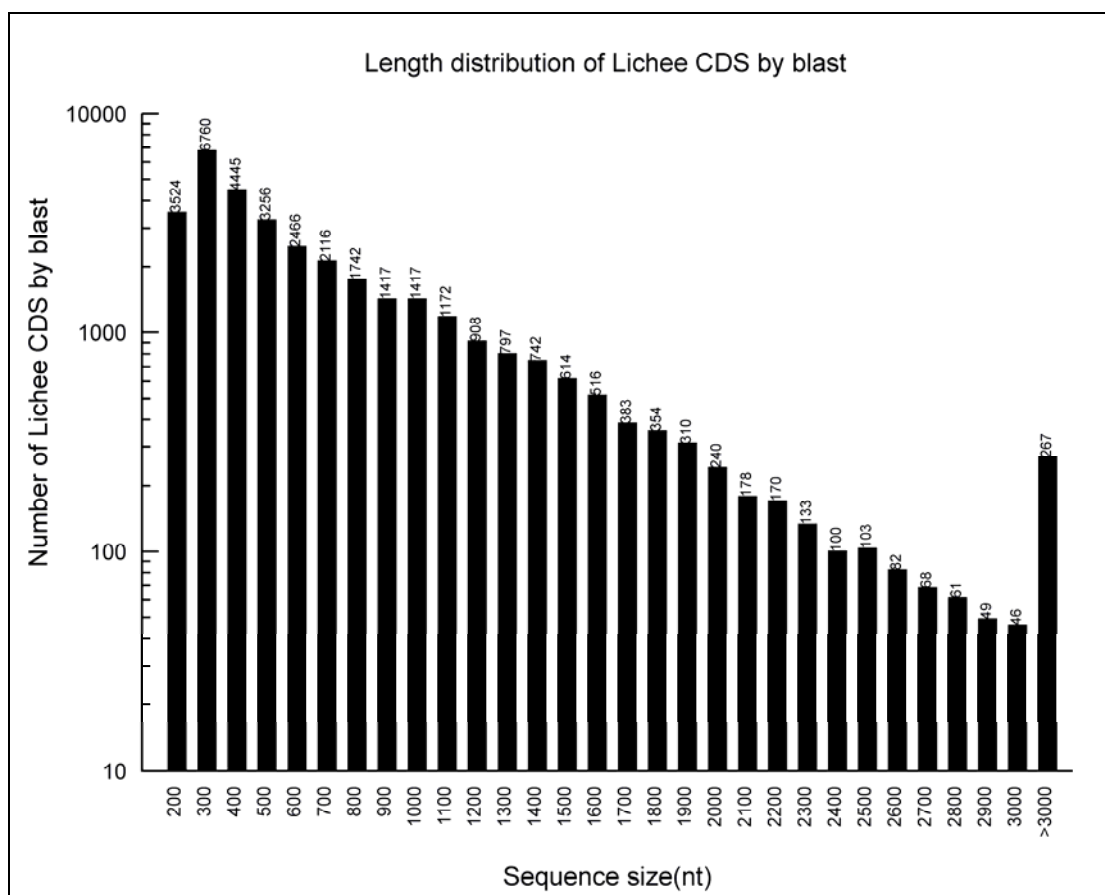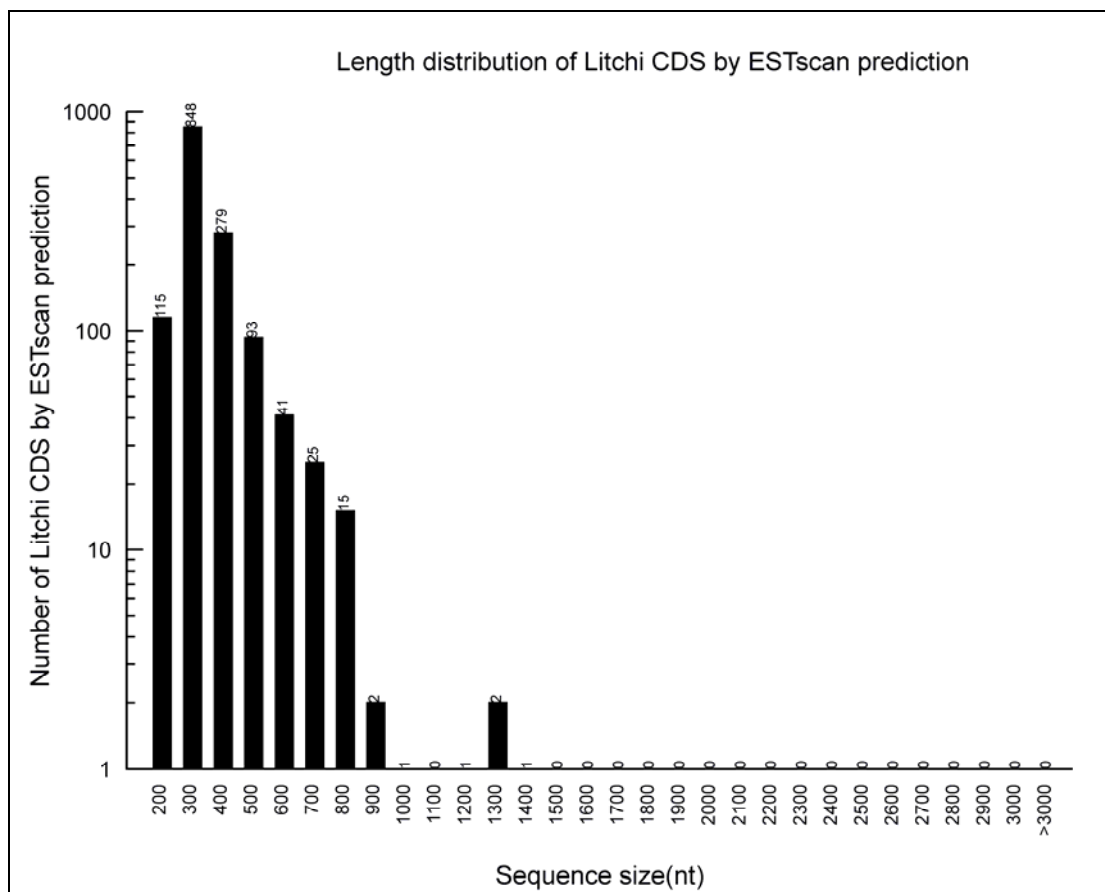

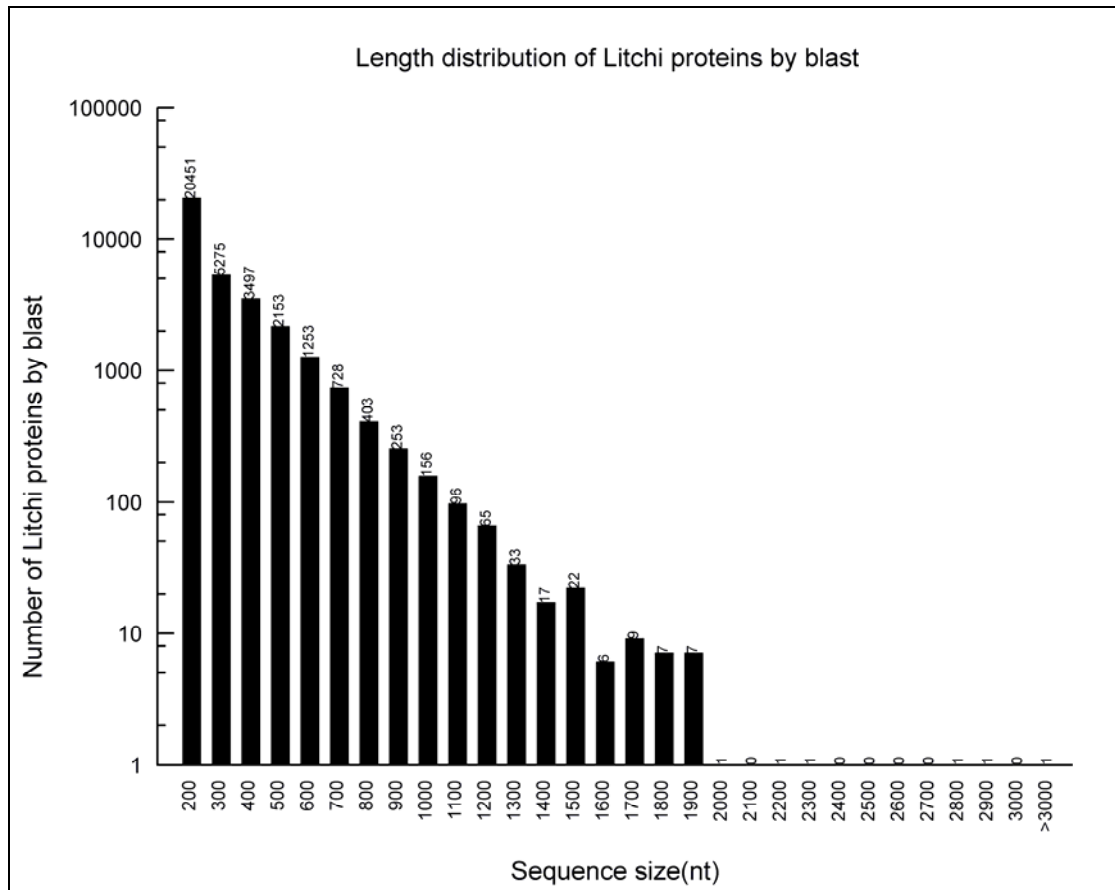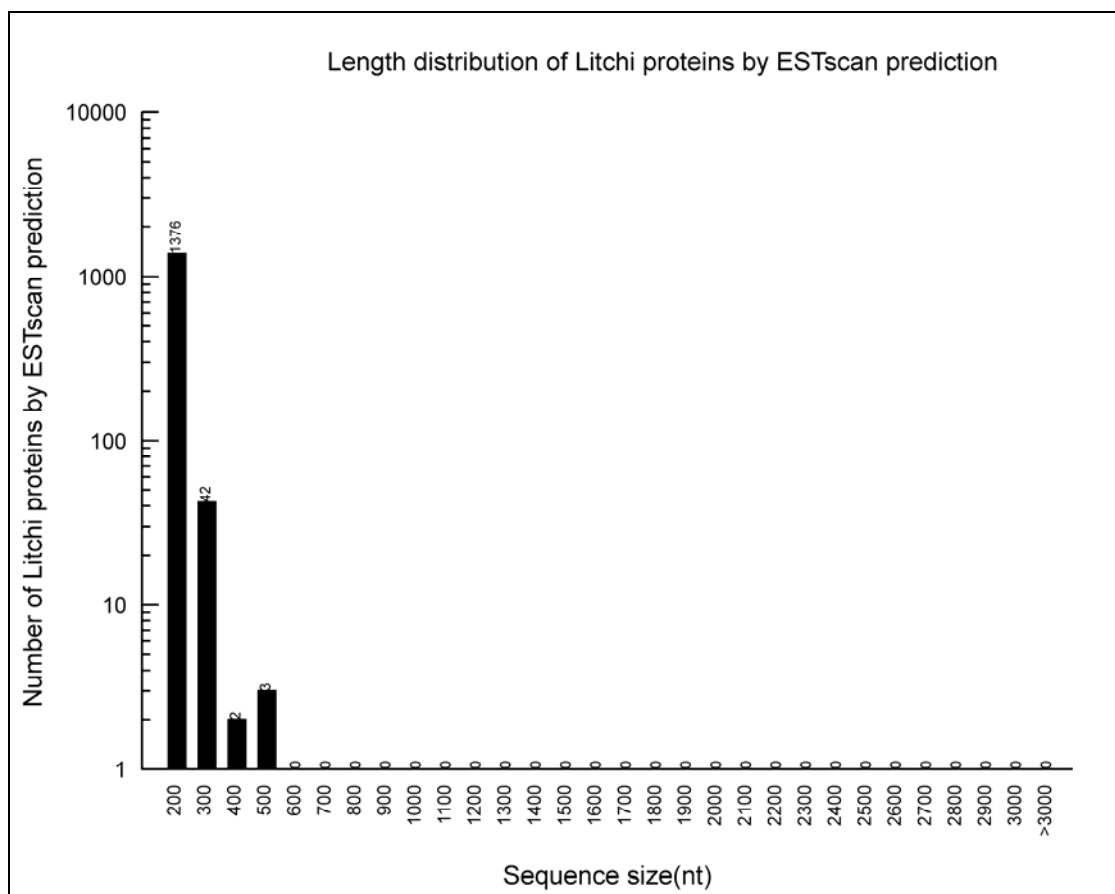

Supplement: Additional file 1: — Length distribution of contigs, unigenes, CDS and proteins from the assembled transcriptome of litchi. [file 12864_2015_1433_MOESM1_ESM.pdf]

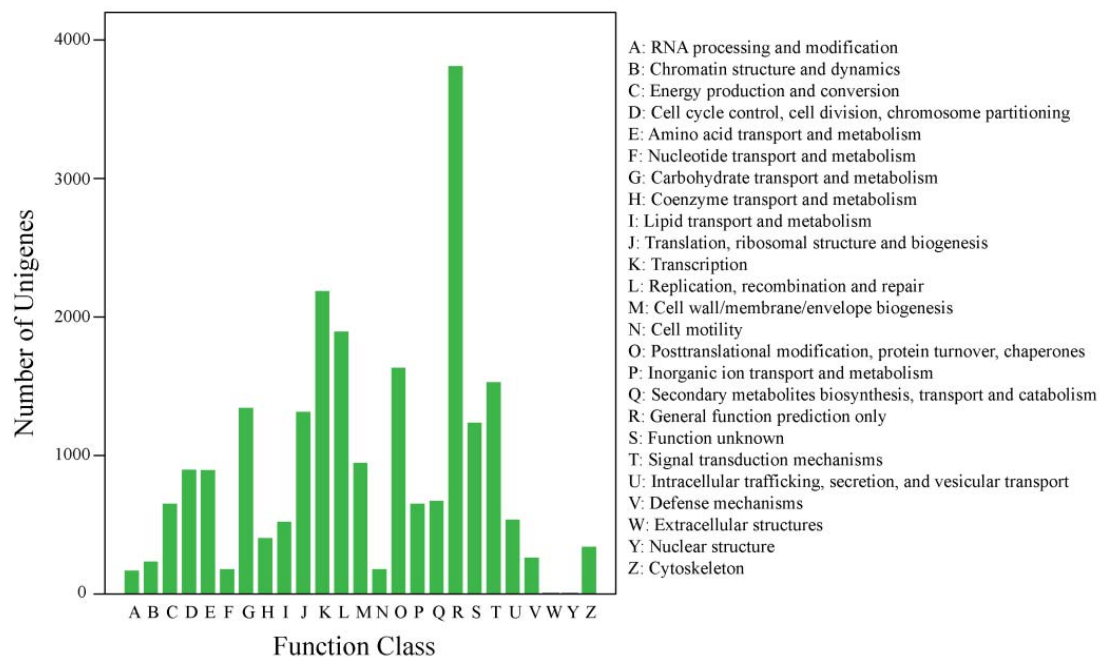

Supplement: Additional file 3: — COG classification of litchi unigenes. [file 12864_2015_1433_MOESM3_ESM.pdf]

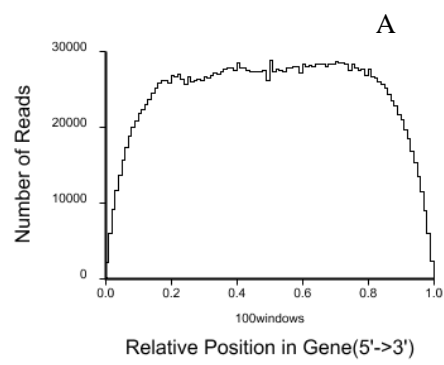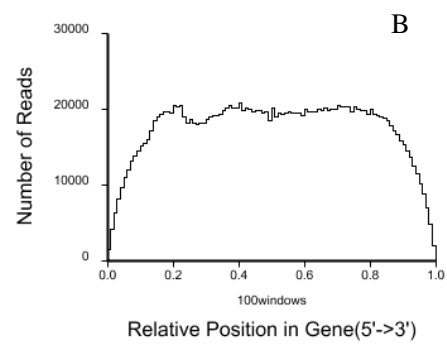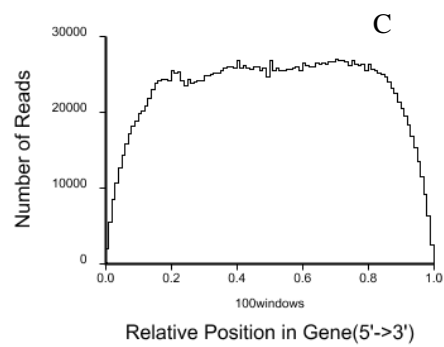

Supplement: Additional file 4: — Distribution of reads on reference genes. Three libraries showed good levels of randomness, with the number of reads evenly distributed over reference genes. A, B and C represent green, yellow and red libraries, respectively. [file 12864_2015_1433_MOESM4_ESM.pdf]

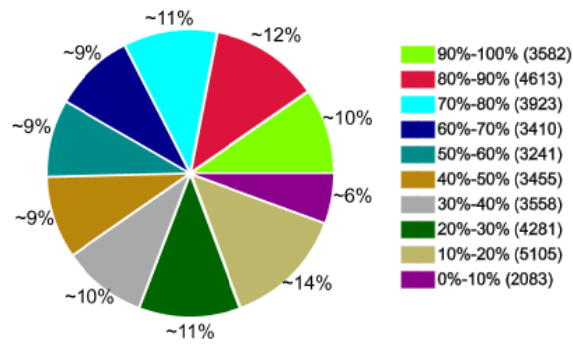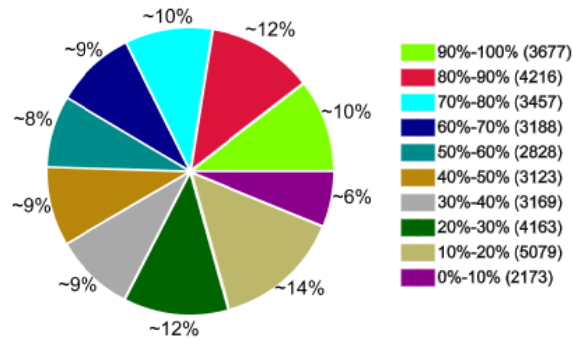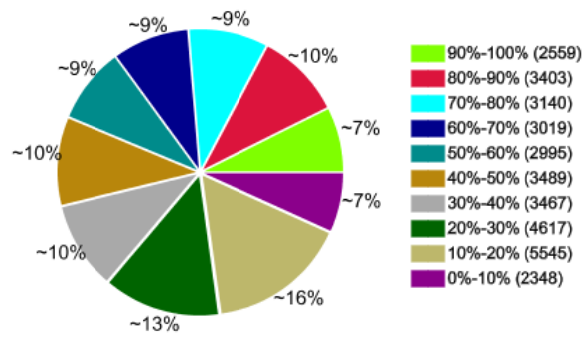

Supplement: Additional file 5: — Percentage of unigenes mapped in the litchi transcriptome data that were expressed at three developmental stages. Gene coverage is the percentage of a gene for which there are matching reads. This value is the ratio of the number of bases in a gene that correspond to unique mapping reads to the total number of bases in that gene. The distribution of unique reads over different read abundance categories show similar patterns for all three RNA-Seq libraries. A, B and C represent green, yellow and red libraries, respectively. [file 12864_2015_1433_MOESM5_ESM.pdf]

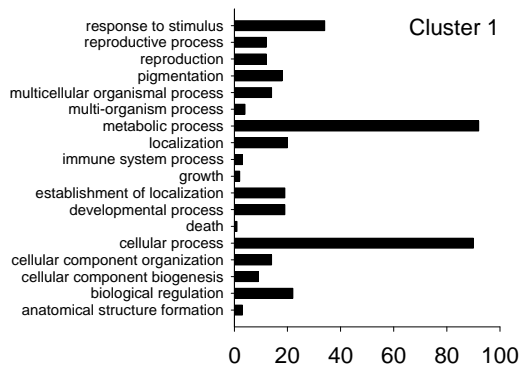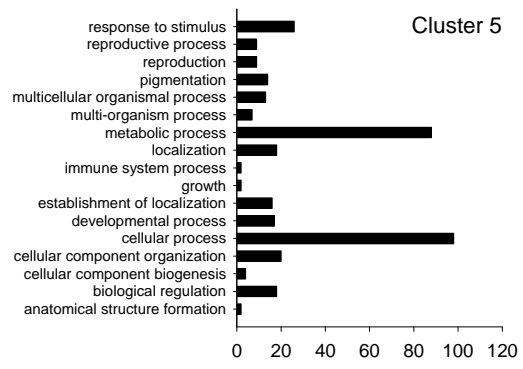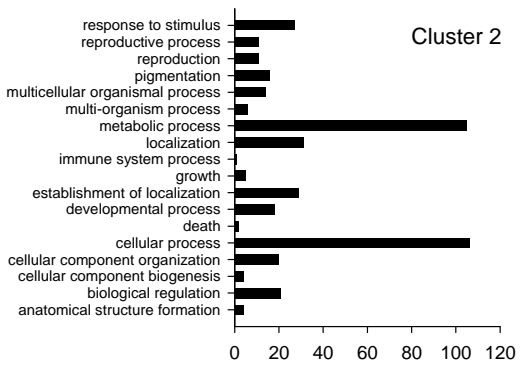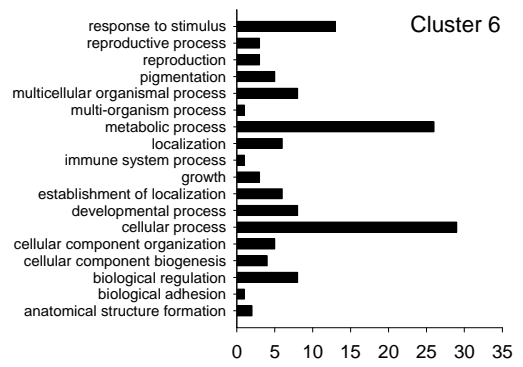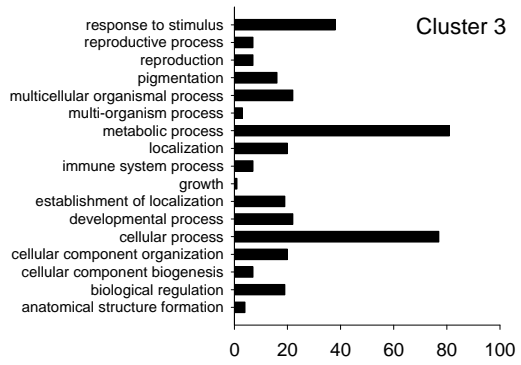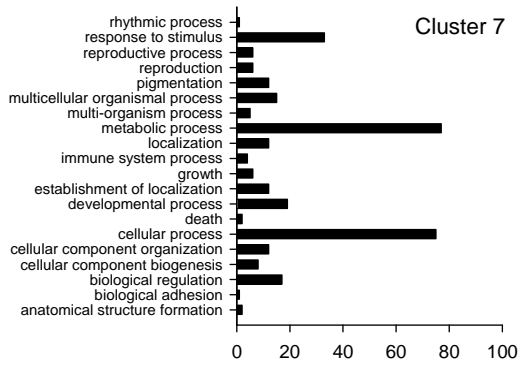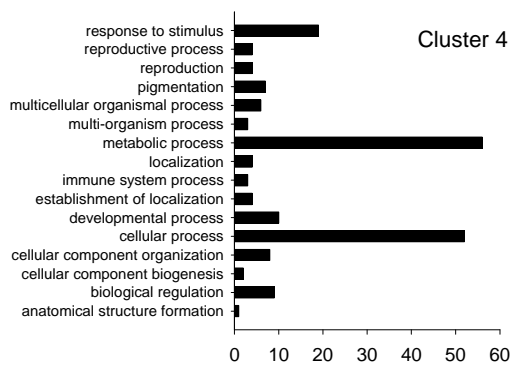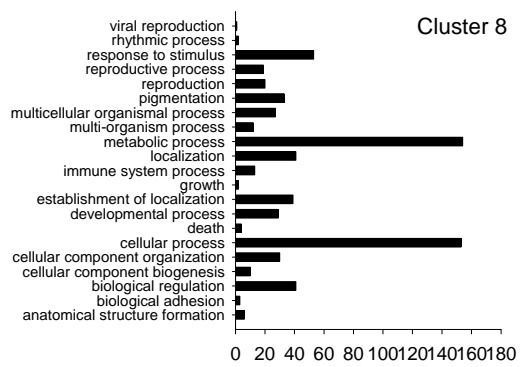

Supplement: Additional file 9: — Histogram representation of the functional categories distribution is expressed as percentage of the amount of genes belonging to the cluster. [file 12864_2015_1433_MOESM9_ESM.pdf]

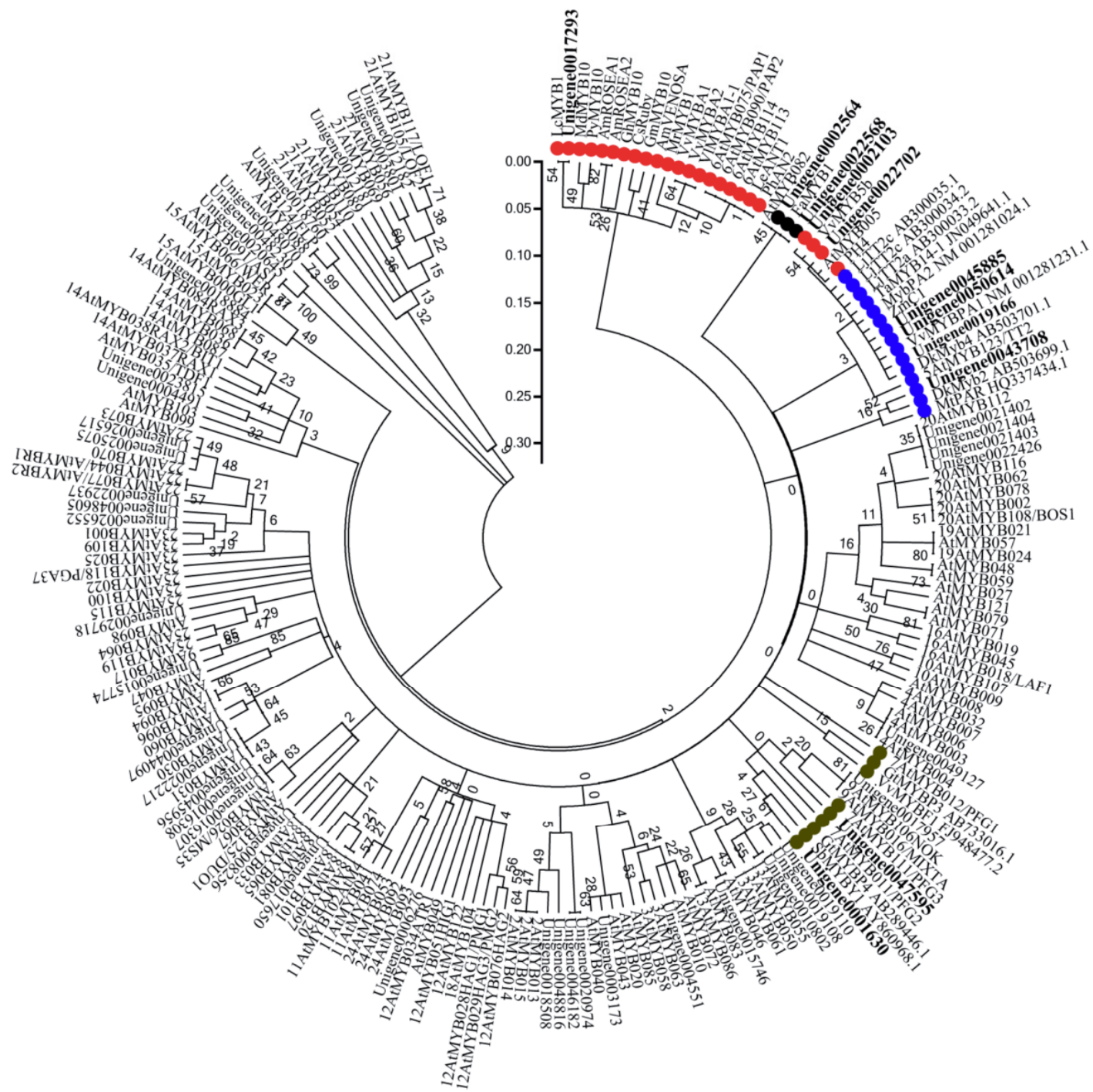

Supplement: Additional file 12: — Schematic representation of the relationships between different R2R3-MYB subgroups. The tree was inferred using the Neighbor-joining method and 1,000 bootstraps with putative amino acid MYB sequences with MEGA 5.0 software. The numbers before the A. thaliana gene ID are subgroup numbers and were designated as previously reported [48]. GenBank protein accession numbers for anthocyanin related MYB transcription factors are listed in Lai et al. [13]. Additional GenBank accession numbers for each gene are as follows: LcSGR, GenBank: KP853137; AtSGR1, GenBank: NP_567673.1; AtSGR2, GenBank: NP_192928.2; AtSGR3, GenBank: NP_564489.1; CaSGR, GenBank: ABX82698.1; GmSGR1, GenBank: NP_001238357.1; GmSGR2, GenBank: NP_001236690.1; HvSGR, GenBank: AAW82955.1; NtSGR, GenBank: ABY19382.1; OsSGR1, GenBank: NP_001063758.1; OsSGR2, GenBank: NP_001054370.1; OsSGR3, GenBank: CAE05787.3; PsSGR, GenBank: A7VLV1.1; SlSGR, GenBank: NP_001234723.1; SbSGR, GenBank: AAW82958.1; ZmSGR1, GenBank: NP_001105770.1; ZmSGR2, GenBank: NP_001105771.1; AdSGR1, GenBank: FG509383; VvMYBPA1, GenBank: NM_001281231.1; DkMyb4, GenBank: AB503701.1; LjTT2c, GenBank: AB300035.1; LjTT2a, GenBank: AB300033.2; LjTT2c, GenBank: AB300034.2; DkMyb2, GenBank: AB503699.1; VvMybPA2, GenBank: NM_001281024.1; MtPAR, GenBank: HQ337434.1; TaMYB14-1, GenBank: JN049641.1; GtMYBP3, GenBank: AB733016.1; GtMYBP4, GenBank: AB289446.1. VvMYBF1, GenBank: FJ948477.2; and SbMYBY1, GenBank: AY860968.1. Arabidopsis thaliana R2R3-MYB family genes were downloaded from the ‘The Database of Arabidopsis Transcription Factors’ (http://datf.cbi.pku.edu.cn/). [file 12864_2015_1433_MOESM12_ESM.pdf]
