# Supplementary material for: Transcriptomic analysis of Litchi chinensis pericarp during maturation with a focus on chlorophyll degradation and flavonoid biosynthesis
Source: BMC Genomics. 2015 Mar 21;16(1):225. doi: 10.1186/s12864-015-1433-4 (PMC4376514; doi:10.1186/s12864-015-1433-4)
Supplement: Additional file 13: — Primers used in real-time PCR analysis to validate expression levels of 15 genes during litchi fruit development. [file 12864_2015_1433_MOESM13_ESM.pdf]

Primers used for quantitative real-time PCR analysis.

Gene	Forward (5′-3′)	Reverse (5′-3′)
Unigene0030141 (F3H)	CTGCAACTCTCACAGCTCTT	GAAGATACCCCAGTCCTCAC
Unigene0004996 (F3'H)	GTGAGATACTGGAGGAGCATAA	TACAGTGCTTGATGTGGTGTC
Unigene0021395 (CHI)	CCTCTATCCTTTTCACACAATC	CGTCTTCAGGCTTCTTATCAT
Unigene0040379 (UFGT)	GCAAGAATGGTACAAGATGAGT	AGCTGTTTCTGCTAGTTGTTTG
Unigene0034194 (PAL)	TAGCTCTACATACCCGTTGATG	CTGTTTGAGATAGGAGCATTGT
Unigene0048046 (LAR)	CAAAGTTGTTGGTACAGTCAAG	AGGATGGTGGTTGTCATAGTAG
Unigene0017070 (ANR)	AGGGCTATGTTGTTCACACTAC	AGCAAAATTGACTGGTGTTG
Unigene0034202 (PAL)	GTCCACCCACTTTCTCTTTTAC	TGACCATTCCTTTCCTGTACT
Unigene0018461 (CHI)	CTTGGAGAAAGTTGTTGAGTTC	GAGGTTATGGTTGTAGGAGACA
Unigene0034195 (PAL)	AGAATTTACGGACCACTTGAC	TAGCCATTGAGGAGATGTTCTA
Unigene0004803 (ANS)	CAGGTAGAAGCCGTAATATGAT	ACATCAGTACCCTCAAAATCAG
Unigene0025384 (CLH)	TCGGTATAGCAGATGAGTTACA	CAGTAGTTCGGTTGACCTAGTCT
Unigene0040467 (CLH)	GACACATGGACGTTTTAGATG	GAGCAACAGAACGGTAGTCTT
Unigene0024586 (PPH)	CGGCTTTGGTGTTGGTAGTT	TTCCCTCCGTTGTATCTCCTT
Unigene0048135 (SGR)	TGGAAAACAAGAGAGAGAGAGA	CCAGAAACAGAACCTTCAACT
ACTIN	ACCGTATGAGCAAGGAAATCACTG	TCGTCGTACTCACCCTTTGAAATC
GAPDH	GATACAGTTCCCGTGTTGTTGAC	CATAAAGACACATAACACCACACTC
